# Clomiphene citrate effect in obese men with low serum testosterone treated with metformin due to dysmetabolic disorders: A randomized, double-blind, placebo-controlled study

**DOI:** 10.1371/journal.pone.0183369

**Published:** 2017-09-08

**Authors:** Carla Pelusi, Vito Angelo Giagulli, Margherita Baccini, Flaminia Fanelli, Marco Mezzullo, Alessia Fazzini, Nicola Bianchi, Matteo Domenico Carbone, Giovanni De Pergola, Marianna Mastroroberto, Antonio Maria Morselli Labate, Renato Pasquali

**Affiliations:** 1 Division of Endocrinology and Center for Applied Biomedical Research, Department of Medical & Surgical Sciences, Alma Mater Studiorum, University of Bologna, S. Orsola-Malpighi Hospital, Bologna, Italy; 2 Outpatients Clinic of Endocrinology and Metabolic Disease, Conversano Hospital, Bari, Italy; 3 Institute of Clinical and Hormonal Research, Foggia, Italy; 4 Nutrition Outpatient Clinic, Clinical Oncology Unit, University of Bari, Bari, Italy; University of Sydney, AUSTRALIA

## Abstract

**Context:**

Low testosterone (T) levels are often found in obese men with impaired glucose tolerance (IGT) and overt type 2 diabetes (T2DM); however, the mechanisms underlying this condition and its correct therapy are still under debate.

**Objective:**

To evaluate the effectiveness of clomiphene citrate (CC) in increasing endogenous T levels in obese men with low serum T and with IGT or T2DM treated with metformin (MET).

**Design:**

Cross-over, randomized, double-blind, placebo-controlled study.

**Methods:**

24 obese men, aged 47.3 ±. 6.3 (range 35–55 years), with low T level (≤3 ng/mL) and naïve diagnosis of IGT or T2DM were included. Subjects were randomized to CC 25 mg/day or placebo (Plac) with MET 2 g/day for 3 months. After a 6-week wash-out period, subjects were moved to the alternative arm for additional 3 months. Clinical evaluation and blood exams performed prior to and at the end of treatment.

**Results:**

Of 24 randomized, 21 were evaluable, classified as IGT (n = 11) or T2DM (n = 10). Compared to baseline levels, T levels increased significantly after 3 months of CC treatment (3.03±0.80 to 5.99±1.67 ng/mL P<0.001) but not after the Plac treatment (2.87±0.78 to 3.09±0.84 ng/mL P<0.001 between the treatments). T changes were similar in IGT and T2DM subjects. Gonadotropins as well raised significantly after CC treatment (LH 3.83±1.45 to 8.53±6.40 mU/mL; FSH 4.84±1.67 to 10.15±5.08 mU/mL P<0.001 respectively), whereas no changes for LH (3.51±1.59 to 3.63±1.39 mU/mL) but a smooth increased for FSH (4.61±2.49 to 5.39±2.65 mU/mL; P = 0.004) were shown after Plac treatment (LH P = 0.001 and FSH P = 0.002 between treatments). Furthermore, fasting glucose (106.8±23.2 to 101.1±25.7 mg/dL; P = 0.004), insulin (19.3±12.1 to 15.6±10.1 μU/mL; P = 0.010) and HOMA-IR (4.94±2.89 to 3.69±2.12; P = 0.001) decreased significantly during the CC treatment period, whereas no significant changes were observed in any of these parameters in the Plac treatment.

**Conclusions:**

A low dose of CC therapy was able to significantly increase serum T levels in all participants with mild modifications of clinical and metabolic parameters.

**Trial registration:**

EudraCT 2011-000439-10

## Introduction

In men, hypogonadism is generally regarded as a clinical condition which might yield and underpin obesity, metabolic syndrome (MS) and even overt type 2 diabetes mellitus (T2DM) [1,2], regardless of where (i.e. at hypothalamic, pituitary and/or testicular levels) and when it onsets (pre-pubertal or post-pubertal onset). On the other hand, men affected by metabolic disorders have low serum testosterone (T) levels [3] whose levels could vary according to individual MS components (i.e. hypertrigliceridemia, abdominal obesity and glycaemia, etc) [4]. Several mechanisms are known to interfere with the hypothalamic-pituitary-gonadal (HPG) axis and thus be responsible for low T level production in obese dysmetabolic patients [5]. Besides the known effect of massive obesity [6] in inducing a partial hypogonadotropic hypogonadism, others factors appear to be involved in causing lowered T levels in this condition, in particular: i) insulin resistance, ii) abnormal adipokine and cytokine release, iii) chronic hypothalamus inflammation [7], and iv) increased estradiol (E2) production [5]. Finally, androgen receptor CAG (AR-CAG) polymorphisms have been shown to play a role in regulating endogenous T levels either in normal men [8] or in obese men with or without T2DM [9].

Beside the well known organic hypogonadism in which a structural defect at hypothalamic/pituitary and/or gonadal levels is present and responsible of the hormonal alterations, the majority of the hypogonadal obese patients may have a functional hypogonadism characterized by low testosterone levels and an intact hypothalamic pituitary testicular (HPT) axis. How to restore normal circulating T levels in these patients is still under debate. Weight loss, whether obtained by life style measures, eventually in association with insulin sensitizer drugs [10–12] or by bariatric surgery [13] has been proven to markedly increase T levels. Studies focused on T replacement therapy (TRT) in obese patients with or without T2DM have proven to be effective in enhancing T deficiency symptoms such as decreased libido and erectile dysfunction [14] and in modulating body composition by increasing lean mass and reducing fat mass whereas the improvement of insulin-resistance and glycemic control is still debated [14,15].

Some new approaches, finalized at restoring endogenous T production in these patients with functional hypogonadism, are represented by aromatase inhibitors or selective estrogen receptor modulator (SERM). In particular, clomiphene citrate (CC), a weak estrogen antagonist belonging to the SERM family, has been shown to improve the hormonal balance in hypogonadotropic hypogonadic patients [16] by enhancing the secretion of gonadotropins and, consequently, of serum T [17].

With this background, we carried out a cross-over randomized controlled study in men presenting with low T blood levels and a new diagnosis of IGT or T2DM, in order to verify whether CC may increase T to normal blood levels and, in this case, to improve metabolic parameters and body fat distribution.

## Materials and methods

The study was a cross-over, randomized, double-blind, placebo-controlled study conducted from December 2011 to February 2016 at two Italian centers, the S. Orsola-Malpighi Hospital (Unit of Endocrinology and Metabolism) of Bologna and the Conversano Hospital (Outpatients Clinic of Endocrinology and Metabolic disease), Bari.

The reason to choose this type of study design, even if complex, was to reduce the influence of confounding factors such as life style (diet and physical activity) and age which may interfere with serum T levels, and to obtain solid results with a small number of subjects selected by mean of strict criteria.

The protocol was approved by the Ethics Committees of the S. Orsola-Malpighi Hospital of Bologna (protocol number UOE/012011) and of the Local Health District of ASL Bari (protocol number 778). Protocol and consent are shown in S1 (original Italian version) and S2 Files (English version).

### Participants

A total of 24 male Caucasian subjects participated in the study. Inclusion criteria were: (i) age between 35 and 55 years; (ii) body mass index (BMI) >30 kg/m^2^; (iii) T levels ≤3 ng/mL (10 nM/L) (measured by electrochemiluminescence immunoassay (ECLIA)); (iv) new diagnosis of IGT or T2DM in accordance with the criteria of American Diabetes Association ADA with glycate hemoglobin (HbA1c) < 8.5% [18]. All patients gave their written informed consent.

Exclusion criteria were as follows: (i) hypogonadism due to organic or genetic causes at hypothalamic-pituitary or gonadal levels detected with clinical, hormonal and radiological examinations, while no genetic tests were needed owing to their clinical history; (ii) IGT or T2DM patients, already on anti-diabetic treatment; (iii) medication interfering with glucose metabolism, including steroid treatment; (iv) patients with any acute or chronic illness that would contraindicate the use of the study medications.

Due to difficulties in recruitment related to the inclusion criteria (*naive* patients) we included in the study 3 subjects with a slightly lower BMI compared to the one requested in the protocol (BMI at baseline of 28.8, 29.6 and 29.7 kg/m^2^, respectively) all of them with T2DM diagnosis. This deviation from the protocol does not have any impact on the aims of the study due to the design of the protocol (cross-over) and subjects’ categorization (still metabolic patients with low T levels). Therefore 12 patients with IGT and 12 T2DM were enrolled in the study.

Age range was arbitrarily defined taking into account the age frequency of the metabolic syndrome [19] and the age physiological decline of T [20].

### Study design and randomization

The study consisted of two-intervention periods (A or B) of 3 months separated by a wash-out period of 6 weeks, based on the CC half-life [17], for a total time of 30 weeks. Patients were randomized into two strata in order to receive either CC at the dose of 25 mg/day (Serofene, Merck Serono, Rome, Italy) or placebo (Plac) in period A and then they shifted to the other treatment in period B. Both drugs were administered in one daily capsule in association with metformin (MET) at the dose of 2 g/day (Glucophage 1000, Bruno Farmaceutici, Rome, Italy). All subjects were asked to follow a 1,600 kcal standardized low calorie diet.

A total of seven clinic visits were organized (V0-V6) (see Table 1), Treatment adherence and any adverse events were collected by the investigators at each visit. At enrollment, the identity of eligible patients was transmitted from both centers to the Clinical Investigation Centre (CIC) of the S. Orsola-Malpighi Hospital of Bologna and the prescription was faxed to the central pharmacy where randomization was assigned according to a list of 24 patients with a 1:1 allocation to either CC or Plac. The list consisted of 4 blocks each with 6 patients in order to balance the randomization for diagnosis (T2DM and IGT) and for center of enrollment. Neither patients, pharmacists, nor investigators knew which treatment was given.

**Table 1:** Outlines of the study design.

Visit	Time	Intervention	Drug delivery	Compliance, efficacy and adverse events	Blood sample
V0	-	Patient enrollment	-	No	Yes
V1	0	Randomization and start of treatment A	CC or Plac and MET	No	Yes
V2	6 weeks	Intermediate visit (A)	CC or Plac and MET	Yes	No
V3	12 weeks	End of treatment A and start of wash-out	-	Yes	Yes
	13–18 weeks	Wash-out period	NO		
V4	18weeks	End of wash-out and start of treatment B	CC or Plac and MET	Yes	Yes
V5	24 weeks	Intermediate visit B	CC or Plac and MET	Yes	No
V6	30 weeks	End of treatment B	-	Yes	Yes

CC: clomiphene citrate; MET: metformin; Plac: placebo

### Endpoints

The primary endpoint of the study was to evaluate the improvement of endogenous T levels from baseline during CC treatment in comparison to Plac. The secondary endpoints were to assess the changes in hormonal and metabolic parameters during the two treatments, as well as to compare hormonal and metabolic parameters between the two subgroups of IGT and T2DM.

### Assessments and measurements

Body weight, height, BMI, and waist circumference were assessed at each visit. Blood samples were drawn at V0, V1, V3, V4 and V6 between 8 and 10 a.m. in the fasted state and prior to the trial medication administration for all measurements. As previously reported, T was measured on at least two occasions with ECLIA (Modular Analytics, E170 System; Roche Diagnostic, GmbH, Mannheim, Germany) only for patient enrollment purposes (V0), whereas all subsequent hormonal assessments, including baseline pretreatment hormones, were performed at V1, V3, V4, and V6 by a validated LC-MS/MS method at the end of the study and used for the efficacy evaluation [21]. The ECLIA T assay displayed a sensitivity of 0.1ng/ml and 7 CV%. The LC-MS/MS assay had a sensitivity of 0.019ng/ml; the imprecision (CV) ranged between 3 and 7% and accuracy between 97 and 100% in the male hypo- and eugonadal range. The ECLIA and the LC-MS/MS assays were compared in a previous study and were reported to have optimal agreement in the male range [21]. E2 and dihydrotestosterone (DHT) were also measured at the end of the study by a recently validated LC-MS/MS method [22] with a sensitivity of 9.8 and 39.1 pg/ml, imprecision between 3 and 6, and between 3 and 10% and accuracy between 96 and 103, and 83 and 101% for E2 and DHT, respectively, in the low and normal male range.

All metabolic parameters were measured at the Central Laboratory of S. Orsola-Malpighi Hospital of Bologna, Italy, as previously reported [23]. Finally, leptin was measured by radioimmunoassay (RIA) kit (HL-81K | human Leptin RIA, Millipore, Germany).

Free testosterone (FT) and free estradiol (FE2) were also calculated according to Vermeulen's formula [24] considering the association constant (Kt) for T equal to 1 x 10^9^ L/mol and the association constant (Ke) for E2 equal to 0.6 x 10^9^ L/mol [25], respectively. The homeostasis model assessment estimate of insulin-resistance (HOMA-IR) was calculated using the formula: fasting glucose (mg/dL)*fasting insulin (μU/mL)/405 [26].

At the beginning of the study, AR-CAG polymorphism was measured in all participants as previously reported [27] to verify whether it could co-regulate either the endogenous baseline T levels or the hormonal response to CC therapy.

### Sample size determination

A value of 2.8 ng/mL was chosen as hypothesized difference of the modification of T between treatments [28,29] in order to obtain physiological hormonal value superior to the inferior limit for hypogonadism definition and with potential clinical relevant outcomes (A mistake occurred in reporting the SD value in the original study protocol; see supporting data with the extended version of the sample size determination). Since no data regarding the within-subject variability of T was available in the literature, the sample size was conservatively overestimated by considering an independent sample design (i.e., by using the within group SD that also takes into account the variability among subjects) instead of a cross-over design (i.e., by using the SD of the changes observed within subjects). In order to simulate the data of a population with the age span of our study, the value of the SD hypothesized for the calculation of the sample size (i.e., 3.0 ng/mL) was estimated as the mean value obtained by weighting 1 and 2 the values of the lower and higher age span groups of the Tenover JS *et al* data [30]. (A mistake occurred in reporting the SD value in the original study protocol; see supporting data with the extended version of the sample size determination).

A sample size of 19 subjects resulted by means of the “PS Power and Sample Size Calculations” package (Department of Statistics, Vanderbilt University, Nashville, TN, USA [31] by considering a power of 80% and a type I error of 5%; thus, we decide to randomize 24 patients in order to allow us to compensate for a possible frequency of drop-out of about 20%. The detailed description of sample size determination is provided in S3 File.

### Statistical analysis

The mean values and standard deviations (SD) of the original data were used as descriptive statistics for scalar variables. The AR-CAG triple repeat number was reported as median value and interquartile range (IQR: 25^th^-75^th^ percentiles) and was compared between IGT and T2DM by means of the linear-by-linear chi square test. The relationship between AR-CAG triple repeat number and hormone blood levels was tested by means of the Spearman rank correlation.

The Shapiro-Wilk’s statistics was applied in order to test scalar variables for the normal distribution and variables that showed distributions significantly different from the normal value were transformed before analysis according to the formula log (x + k) since all of them had highly significant positive skewness coefficients (P≤0.003) at the z-test. The values of the constant coefficients (k) that zeroed the skewness coefficients were chosen in the transformation functions. All variables showed normal distribution after transformation (P≥0.291).

Baseline characteristics of the subgroups with IGT or T2DM were compared by means of the one-way analysis of variance (ANOVA) while, in order to analyze the endpoint data according to the study design, the 4-way repeated measure ANOVA was applied by considering two intra-subject factors and two inter-subject factors. The two intra-subject factors were: i) the effects of the two treatments (they were estimated by comparing post-treatment *vs*. pre-treatment values); ii) the efficacy of the addiction of CC to Met (it was evaluated by means of the comparison between treatments, i.e., CC *vs*. Plac. The two inter-subject factors were: i) the effect of different glycemic status (it was estimated by comparing T2DM *vs*.IGT patients); ii) the cross-over effect (i.e., the possible role of the sequence of administration of the two treatments; it was evaluated by comparing the subgroup of patients receiving CC in period A and then Plac in period B with the subgroup of patients receiving Plac in period A and then CC in period B). Nested designs were used in the 4-way ANOVAs in order to test the effect of a factor within the categories of the other factors. Since no multiple level factors were present in the experimental design and an unique multi-way ANOVA was made for each of the dependent variable, no post-hoc analyses were made, as well as all effect estimates were based on a unique residual; thus, the problem of multiple comparisons did not arise. The data were managed and analyzed by using the IBM SPSS Statistics package (version 23; IBM, Co., Armonk, NY, USA) and two-tailed P values less than 0.05 were considered statistically significant.

## Results

### Study subjects and baseline characteristics

**Fig 1:**
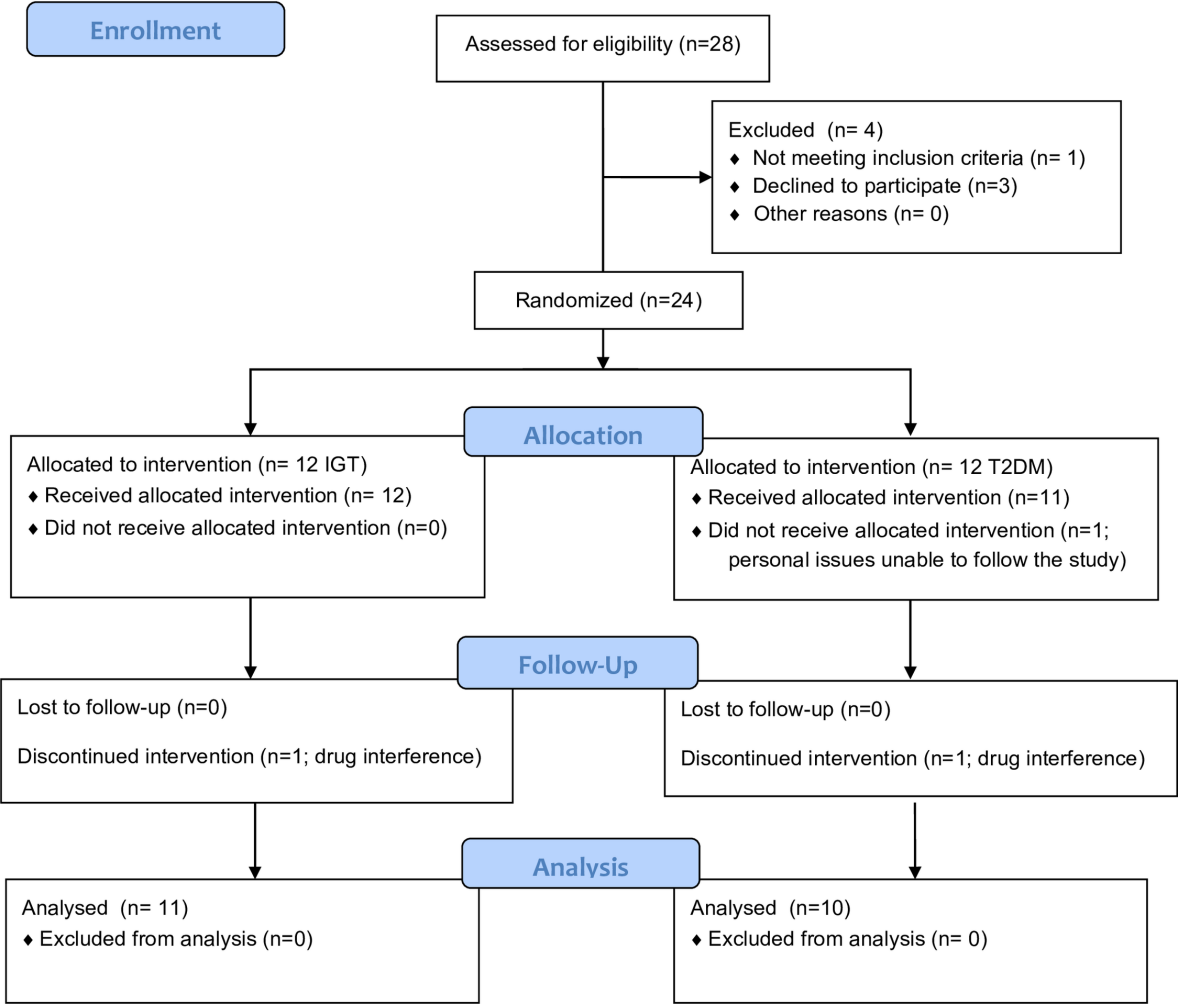
Study flowchart. Flowchart describing the number of subjects assessed for eligibility, included in the randomization and completed the study.

A flow-chart of the patients enrolment is reported in Fig 1. Basal data of all the 24 enrolled patients according to metabolic state (IGT and T2DM) showed that, in accordance with the metabolic state, age (P = 0.006), fasting glucose (P = 0.001) and HbA1c levels (P = 0.013) were significantly different between IGT and T2DM, while no significant differences were observed between the two subgroups both in the main clinical and hormonal parameters as well as in the AR-CAG number (Table 2). Interestingly, in the overall population, the AR-CAG triple repeat number was not significantly related with hormone blood levels (T LC/MS-MS, P = 0.426; FT, P = 0.106; DHT, P = 0.385; E2, P = 0.864; FE2, P = 0.894; SHBG, P = 0.647; LH, P = 0.284; FSH, P = 0.738). At variance, the AR-CAG triple repeat number showed a positive correlation with FT in T2DM patients (P = 0.027) but not in those with IGT.

**Table 2:** Baseline clinical and biochemical characteristics of all subjects and according to their metabolic state (IGT and T2DM).

	All subjects (n = 24)	IGT (n = 12)	T2DM (n = 12)	P ^b^
Age (years)	47.3±6.3	44.0±6.4	50.7±4.2	**0.006**
BMI (kg/m^2^)	35.3±5.4	37.1±6.1	33.5±4.0	0.116
Waist circumference (cm)	116±11	120±12	111±9	0.078
T (ng/mL)	2.33±0.48	2.30±0.48	2.35±0.50	0.812
LH (mU/mL)	3.56±1.59	3.89±1.73	3.23±1.43	0.546
FSH (mU/mL)	4.50±2.04	4.39±2.55	4.61±1.49	0.397
Fasting glucose (mg/dL)	110±23	96±14	125±22	**0.001**
Fasting insulin (μU/mL)	20.3±11.8	24.7±12.3	15.5±9.6	0.085
HbA1c (%)	5.96±0.62	5.66±0.36	6.27±0.69	**0.013**
AR-CAG number ^a^	20.5 (16.0–22.0)	21.0 (18.5–23.5)	17.5 (16.0–22.0)	0.183 ^c^

The data are expressed as mean±SD. AR: androgen receptor; BMI: body mass index; FSH: follicular stimulating hormone; HbA1c: glycosylated hemoglobin; LH: luteinizing hormone; T: testosterone.

^a^ Median (interquartile range; IQR)

^b^ One-way analysis of variance (ANOVA)

^c^ Linear-by-linear chi square test

Only 21 out of the 24 enrolled subjects went on to receive both treatments and concluded the study. In fact, three subjects dropped out and were excluded from the analysis: two of them because of drug interference during the treatment period (one T2DM patient started steroid treatment for chronic obstructive pulmonary disease; a second IGT patient had an injection of HCG) and, finally, a third one with T2DM refused to start the treatment for personal issues after randomization. Of the remaining 21 patients included in the study, 11 had IGT and 10 had T2DM.

### Hormonal changes in all subjects and according to their metabolic state

The hormonal values before and after each treatment are shown in Table 3. During the CC treatment serum T (P<0.001), FT (P<0.001), DHT (P<0.001), E2 (P<0.001), FE2 (P<0.001), SHBG (P = 0.001) and both gonadotropins (LH: P<0.001 and FSH: P<0.001) increased significantly from baseline in the overall population, regardless of their metabolic condition (IGT or T2DM). By contrast, during Plac treatment, no significant differences were observed in any of the hormonal levels except for an increase in FSH (P = 0.004) and FT (P = 0.034). Consequently, when the mean differences of all hormonal levels between the two treatment periods in the whole cohort were compared, the rise in all hormones (T, FT, E2, FE2, P<0.001 respectively; DHT and LH, P = 0.001; SHBG P = 0.009; FSH P = 0.002) was significantly much higher in the CC treatment group with respect to the Plac group (Table 3).

**Table 3:** Hormonal values before and after each treatment period in all subjects and according to their metabolic state (IGT or T2DM).

	Clomiphene Citrate			Placebo			P (Δ)
	Pre- treatment	Post- treatment	P	Pre- treatment	Post- treatment	P	
**T LC/MS-MS (ng/mL)**							
**All subjects**	3.03±0.80	5.99±1.67	**<0.001**	2.87±0.78	3.09±0.84	0.070	**<0.001**
IGT	2.98±0.75	6.53±1.74	**<0.001**	2.98±0.69	3.13±0.84	0.557	**<0.001**
T2DM	3.09±0.88	5.39±1.43	**<0.001**	2.74±0.88	3.05±0.88	0.051	**0.005**
**FT (pg/mL)**							
**All subjects**	74.4±22.9	149.6±49.3	**<0.001**	71.3±24.9	78.4±23.2	**0.034**	**<0.001**
IGT	74.2±20.5	165.7±54.6	**<0.001**	76.2±20.1	76.9±21.1	0.954	**<0.001**
T2DM	74.7±26.4	132.0±37.8	**<0.001**	65.9±29.4	80.1±26.4	**0.005** ^a^	0.050
**DHT (ng/mL)**							
**All subjects**	0.173±0.088	0.287±0.148	**<0.001**	0.188±0.126	0.181±0.098	0.860	**0.001**
IGT	0.160±0.076	0.280±0.151	**<0.001**	0.170±0.156	0.154±0.065	0.701	**0.008**
T2DM	0.185±0.101	0.296±0.151	**0.003**	0.208±0.085	0.208±0.121	0.509	**0.008**
**E2 (pg/mL)**							
**All subjects**	23.3±7.8	48.6±20.2	**<0.001**	25.2±6.2	23.7±9.1	0.304	**<0.001**
IGT	26.1±7.5	57.0±22.4	**<0.001**	26.0±6.8	28.1±10.2	0.442	**0.001**
T2DM	20.3±7.3	39.2±12.9	**<0.001**	24.0±5.6	19.3±5.1	0.060	**<0.001**
**FE2 (pg/mL)**							
**All subjects**	0.319±0.114	0.610±0.223	**<0.001**	0.352±0.094	0.328±0.122	0.517	**<0.001**
IGT	0.350±0.102	0.705±0.216	**<0.001**	0.358±0.088	0.374±0.127	0.673	**<0.001**
T2DM	0.285±0.122	0.504±0.187	**<0.001**	0.342±0.110	0.281±0.102	0.241	**<0.001**
**SHBG (nmol/L)**							
**All subjects**	22.8±11.1	26.2±10.0	**0.001**	22.4±8.3	22.5±8.3	0.882	**0.009**
IGT	23.1±13.7	26.4±12.4	**0.012**	21.3±7.3	23.5±8.1	0.067	0.477
T2DM	22.4±8.1	26.1±7.2	**0.007**	23.7±9.6	21.3±8.9	0.053 ^a^	**0.003**
**LH (mU/mL)**							
**All subjects**	3.83±1.45	8.53±6.40	**<0.001**	3.51±1.59	3.63±1.39	0.806	**0.001**
IGT	4.60±1.43	10.69±7.77	**<0.001**	4.15±1.57	4.17±1.52	0.959	**0.006**
T2DM	2.99±0.94	6.15±3.47	**0.001**	2.81±1.35	3.04±0.97	0.697	**0.027**
**FSH (mU/mL)**							
**All subjects**	4.84±1.67	10.15±5.08	**<0.001**	4.61±2.49	5.39±2.65	**0.004**	**0.002**
IGT	4.45±1.76	10.77±5.41	**<0.001**	4.75±2.65	5.10±2.73	0.324	**<0.001**
T2DM	5.27±1.55	9.46±4.89	**0.008**	4.47±2.44	5.70±2.66	**0.002**	0.334

The data are expressed as mean±SD; P values represent the intra-treatment differences and P(Δ) values represent the comparison of the changes between treatments. All subjects in addition were taking metformin, 2g/day

^a^ P<0.05: T2DM *vs*. IGT

Intriguingly, some difference was found between the IGT and T2DM subgroups during treatments. In IGT, in fact, during CC treatment, all hormones (all P<0.001) and SHBG (P = 0.012) significantly increased from baseline; whereas no significant change was observed in the Plac group (see Table 3). Similar findings for most parameters were present in patients with T2DM, serum T, FT, E2, FE2 (all P<0.001), DHT (P = 0.003), SHBG (P = 0.007), LH (P = 0.001) and FSH (P = 0.008) levels during CC treatment being significantly increased, whereas in the Plac treatment group no significant change occurred in any of the parameters, except for an increase in FT (P = 0.005) and FSH (P = 0.002) levels. Comparing the two treatments, the increase in all hormones was higher during the CC treatment, with the exception, only in T2DM, of FT and FSH levels, due to the fact that both hormones significantly increased and similarly in both treatments (see Table 3).

Finally, we evaluated differences in the hormonal response between IGT and T2DM. Similar changes were shown in all parameters during the CC treatment, although a higher increase in FT and a significant reduction of SHBG levels in the T2DM vs IGT in the Plac treatment was detected (Table 3).

### Metabolic changes in all subjects and according to their metabolic state

Clinical characteristics and metabolic parameters in all participants and in the two subgroups (IGT and T2DM) at baseline and after the two treatments are shown in Table 4. In all subjects, during the CC treatment, there was a tendency towards a decrease in both BMI and waist circumference (P = 0.058 and P = 0.066, respectively), and a significant reduction of fasting glucose (P = 0.004) and insulin (P = 0.010) levels, and in the HOMA-IR (P = 0.001), whereas no significant changes occurred in either HbA1c or leptin levels. At variance, no significant variations in any parameters were detected during the Plac treatment. Furthermore, after comparing the mean changes of all clinical and metabolic parameters between the two treatments, no significant differences were found. We then examined the two subgroups separately. In the IGT, a significant improvement of BMI (P = 0.013), waist circumference (P = 0.037), fasting glucose (P = 0.025) and insulin (P = 0.005) and HOMA-IR (P = 0.001) was observed after the CC treatment. By contrast, only a small but significant improvement in fasting glucose levels (P = 0.042) occurred in the T2DM. Notably, neither clinical nor metabolic parameters improved after the Plac treatment in either IGT or T2DM subjects. The comparison between changes during the two treatments in all patients considered together and the two subgroups considered separately (IGT and T2DM) did not reveal any significant difference.

**Table 4:** Clinical characteristics and metabolic parameters before and after each treatment period in all subjects and according to their metabolic state (IGT or T2DM).

	Clomiphene Citrate			Placebo			P (Δ)
	Pre- treatment	Post- treatment	P	Pre- treatment	Post- treatment	P	
**BMI (kg/m**^**2**^**)**							
**All subjects**	35.6±5.9	35.2±5.7	0.058	35.4±5.6	35.1±6.2	0.144	0.882
IGT	37.6±6.8	36.7±6.5	**0.013**	37.2±6.1	37.1±7.3	0.258	0.573
T2DM	33.4±4.0	33.5±4.2	0.863	33.4±4.5	33.0±4.1	0.332	0.459
**Waist (cm)**							
**All subjects**	116.5±12.3	115.4±13.1	0.066	115.9±10.9	115.4±11.5	0.437	0.348
IGT	121.2±14.8	119.5±16.6	**0.037**	119.4±12.7	119.6±13.5	0.942	0.055
T2DM	111.4±6.1	110.9±6.0	0.582	112.0±7.2	110.8±6.8	0.258	0.537
**Fasting glucose (mg/dL)**							
**All subjects**	106.8±23.2	101.1±25.7	**0.004**	106.6±23.5	100.2±22.3	0.133	0.901
IGT	94.5±13.0	89.0±13.7	**0.025**	95.0±15.6	91.8±12.2	0.690	0.452
T2DM	120.3±24.9	114.5±29.7	**0.042**	119.4±24.6	109.5±27.6	0.090	0.583
**HbA1c (%)**							
**All subjects**	5.88±0.69	5.86±0.76	0.445	5.89±0.60	5.80±0.64	0.284	0.376
IGT	5.56±0.41	5.54±0.41	0.847	5.63±0.41	5.53±0.38	0.443	0.470
T2DM	6.24±0.78	6.22±0.90	0.688	6.17±0.68	6.10±0.76	0.448	0.590
**Insulin (µU/mL)**							
**All subjects**	19.3±12.1	15.6±10.1	**0.010**	18.7±13.0	16.2±10.0	0.815	0.217
IGT	24.6±13.9	18.3±11.9	**0.005**	24.5±13.4	20.2±11.6	0.324	0.419
T2DM	13.4±6.3	12.6±7.1	0.365	12.3±9.3	11.9±5.6	0.529	0.341
**HOMA-IR**							
**All subjects**	4.94±2.89	3.69±2.12	**0.001**	4.67±3.20	3.93±2.46	0.626	0.213
IGT	5.70±3.15	4.00±2.57	**0.001**	5.82±3.52	4.65±2.93	0.348	0.383
T2DM	4.10±2.46	3.34±1.55	0.101	3.41±2.37	3.14±1.61	0.817	0.374
**Leptin (ng/mL)**							
**All subjects**	17.0±11.3	15.3±10.7	0.489	17.4±15.3	15.8±13.2	0.722	0.760
IGT	22.5±12.1	17.6±10.8	0.101	24.6±17.5	21.5±15.4	0.777	0.487
T2DM	10.9±6.7	12.8±10.5	0.474	9.5±6.6	10.1±7. 6	0.646	0.789

The data are expressed as mean ±SD; P values represent the intra-treatment differences and P(Δ) values represent the comparison of the changes between treatments. All subjects were taking metformin 2g/day.

### Sensitivity analysis

The sensitivity analysis was performed by excluding the 3 patients with BMI below 30 kg/m^2^. Only the main results of the study were analyzed (hormonal profile: T, LH, FSH; metabolic profile: fasting glucose, insulin, and HOMA-IR). The P values of the results of this analysis are reported in Table 5. These P values were very similar when compared with those evaluated in the entire population (Table 3 for hormonal profile and Table 4 for metabolic profile) because all these three patients had baseline BMI extremely near to the cut-off value for detecting obesity (28.8, 29.6 and 29.7 kg/m^2^) although all three were T2DM patients. The only difference observed in the hormonal profile was the LH levels found in the comparison between the two treatment in the T2DM patients (a P value near the significant limit (P = 0.078) found in the sensitivity analysis instead of the significant value found in the entire T2DM population (P = 0.027)). As far as the metabolic profile is concerned, the change of glucose fasting levels after Plac treatment became significant both in all subjects and in T2DM patients (sensitivity analysis: P = 0.039 and P = 0.021, respectively; entire population: P = 0.133 and P = 0.090, respectively), whereas the change of insulin fasting levels observed after CC treatment in all subjects failed to reach the significant level (sensitivity analysis: P = 0.116; entire population: P = 0.010).

**Table 5:** Sensitivity analysis performed after excluding the 3 patients with BMI below 30 kg/m^2^.

	Effect of Clomiphene Citrate (CC)	Effect of Placebo (Plac)	Comparison CC *vs*. Plac P (Δ)		Effect of Clomiphene Citrate (CC)	Effect of Placebo (Plac)	Comparison CC *vs*. Plac P (Δ)
**T LC/MS-MS:**				**Fasting glucose:**			
**All subjects**	**<0.001**	0.122	**<0.001**	**All subjects**	**0.000**	**0.039**	0.848
IGT	**<0.001**	0.596	**<0.001**	IGT	**0.002**	0.682	0.399
T2DM	**0.001**	0.115	**0.028**	T2DM	**0.001**	**0.021**	0.361
**LH:**				**Insulin:**			
**All subjects**	**<0.001**	0.773	**0.004**	**All subjects**	**0.116**	0.625	0.741
IGT	**<0.001**	0.929	**0.006**	IGT	**0.003**	0.351	0.429
T2DM	**0.015**	0.765	0.078	T2DM	**0.491**	0.900	0.833
**FSH:**				**HOMA-IR:**			
**All subjects**	**<0.001**	**0.017**	**0.002**	**All subjects**	**0.012**	0.400	0.710
IGT	**<0.001**	0.262	**0.001**	IGT	**0.001**	0.370	0.388
T2DM	**0.030**	**0.024**	0.210	T2DM	0.791	0.713	0.828

Only the main results of the study were analyzed (hormonal profile: T, LH, FSH; metabolic profile: fasting glucose, insulin, and HOMA-IR). The P values of the effects of Clomiphene Citrate (CC) and Placebo (Plac) are reported in the Table together with their comparison (P (Δ)). These values should be compared with those evaluated in the entire population (hormonal profile: Table 3; metabolic profile: Table 4).

### Cross-over effect between treatment periods

We investigated potential carry-over effects on hormonal and metabolic parameters by comparing the two strata of all patients with different treatment sequences (CC and Plac vs. Plac and CC). No significant differences were shown for T, FT, E2 and FE2 between the two treatment periods, implying that there were no drug carry-over effects on sex hormones. HOMA-IR decreased significantly more in those subjects that were given CC in the first period compared to those that had this treatment in the second period of the study (P = 0.038), although neither BMI nor HbA1c progressively decreased at any time during the study. Furthermore, patients who were given CC in the second period of the study started with higher SHBG levels (P = 0.002) and ended with higher SHBG (P = 0.011) and LH concentrations (P = 0.013) compared to those who had this drug combination in the first period.

### Safety of treatment

CC was well tolerated and there were no complaints of any adverse event throughout the study.

## Discussion

This is the first study showing that CC is able to significantly improve endogenous T secretion, reaching physiological levels in obese dysmetabolic patients with low serum T concentrations, regardless of their glucose tolerance state (IGT or T2DM), on MET treatment. The IGT and T2DM subjects differed, at baseline, only in glucose and HbA1c levels and in age, which is consistent with the classification of these patients and the natural history of this metabolic disease.

The efficacy of CC in improving T endogenous secretion at several dosages and for different periods has been reported in several cohorts of male patients characterized by infertility and/or hypogonadotropic hypogonadism [16,32–34], and those data were recently confirmed by a meta-analysis of randomized controlled trial where the estrogen antagonists therapy were given to men with idiopathic male infertility [35]. All studies showed a successful increment of T levels in association with an increase in gonadotropins without specific side-effects [36], supporting the potential use of CC in restoring the HPG axis in hypogonadal patients with conserved testis function. The present study further supports the concept that the T increase during the CC treatment was associated with the elevation of serum LH and FSH, which is in agreement with the antiestrogenic activity at hypothalamic-pituitary levels of CC [15]. As a matter of fact, during CC administration, besides the rise in serum T levels, there was an increase in both E2 and particularly of DHT, the active metabolites deriving from the intra-testicular and peripheral activity of aromatase [37] and 5a-reductase, respectively [38]. Notably, these positive hormonal changes were detected only during CC administration, even if a modest increment of FT and unexpectedly of FSH was reported during the Plac treatment. Previous works have shown that diet alone or in association with MET is able to progressively improve androgens and FSH levels in obese subjects with metabolic syndrome, according to the amount of weight loss [8–10]; however these results are difficult to obtain and therefore not always sufficient in reaching physiological hormonal levels. Even if the existing literature presents some contradictory data on the effect of MET on testis functions [39,40], our findings are in accordance with studies carried out in diabetic rats in which MET was shown to inhibit the diabetes-induced damages in testicular tissue, increasing plasma levels of T, LH and FSH as well as reducing Sertoli cell [41] and germ cell apoptosis [42].

This hormonal profile further prove that most obese men with T2DM are characterized by a state of functional hypogonadotropic hypogonadism [5] rather than an organic defect or an hypergonadotropic hypogonadism condition that may respond to life style measures with medications facilitating the endogenous production of T, such as SERM.

Conversely, the increment of SHBG levels observed during the CC treatment may reflect a direct estrogen stimulating production of that protein at hepatic levels, possibly combined with a mild effect obtained by insulin-resistance improvement shown in this phase.

During treatment with CC, the improvement of all parameters of the glucose status, as well as the decrease in BMI and waist circumference observed in the IGT patients but not in those with T2DM, may be largely attributed to the still preserved insulin secretion, and to the changes in body weight and visceral fatness. Therefore, the mechanisms responsible for the amelioration of glucose metabolism in these subjects appear to depend on different factors partly related to changes in the androgen status but also to body composition and insulin secretory reserve.

We measured leptin because it is known to be differently modulated by sex hormones [43]. However, there was no difference in serum leptin levels in any participants in spite of specific differences in the amount of body fat, particularly relevant for patients with IGT. These data may be consistent with the coordinated increase in both T and E2 levels, especially during CC treatment, precluding the prevalent action of one of them. On the other hand, the previous interpretation by some authors that T lowers serum leptin levels merits further investigation, at least in men.

The sensitivity analysis performed after excluding the three T2DM subjects with BMI under 30 kg/m2 showed similar hormonal changes during treatments and a close metabolic variations with the only exception of basal insulin levels, further supporting the efficacy of CC in these subjects.

No treatment carry-over effects on hormonal levels and metabolic parameters were observed. We only found a mild metabolic improvement in the group that were given CC in the first period as well as a higher increment in SHBG and LH levels in the group that took CC in the second phase, further supporting a role of CC in association with life style modification and MET administration in ameliorating metabolic parameters probably through the increment of sex hormones. In addition, only a positive correlation was shown between CAG polymophism with FT in the T2DM subgroup at baseline, suggesting its possible modulating role in HPG axis in these patients [6,8].

Our study has a number of strengths including (i) its cross-over design, (ii) the absence of carry-over effects; (iii) the lack of drop out due to the treatment, (iv) the separate evaluation of specific subgroups of patients (IGT or T2DM) and, finally, (v) the measurement of steroids by LC/MS-MS. In particular, the beneficial effect of CC when added to MET alone resulted in an increase of T equal to 2.74 ng/mL, i.e. a value extremely near to the difference hypothesized for the sample size determination (2.8 ng/mL), thus confirming the efficacy of the treatment observed in the present study and its potential clinical relevance. Moreover, this is the first study performed in male subjects with the administration of CC in association with MET. On the other hand, one limitation of our study might be the short treatment period that is likely to have precluded the lack of substantial differences in the anthropometric and metabolic parameters during the two phases of the study. In fact, it could be speculated that a longer treatment phase, such as six months or more, would have achieved more significant changes in body composition and in metabolic parameters that only are mildly evident at three months. Moreover, it is possible that the administration of MET during all the study period may have hidden the metabolic effect of CC.

Therefore, in these patients CC can be considered an alternative to TRT that has been shown to have several health risks, including an increased risk of thrombosis or stroke, heart disease and prostate problems, possibly due to supra-physiological doses of T [44,45]. Furthermore, there is no doubt that even long-term treatment with CC, in case associated with MET, is both less expensive than and as effective as the TRT [46].

In conclusion, CC therapy added to MET, appears to represent a valid alternative treatment to classic TRT in dysmetabolic obese men with low T serum levels and intact HPT axis, since it restores a normal sex hormone profile and is associated with some metabolic improvement, particularly on glucose metabolism, and, possibly, on insulin sensitivity.

## Supporting information

S1 File Protocol UOE.01-2011. Original (Italian) version of the protocol.
(PDF)

S2 File English version protocol UOE.01-2011. English version of the protocol.
(PDF)

S3 File Sample size PO. Detalied explanation of the sample size determination.
(DOC)

S4 File CONSORT 2010 checklist new. (PDF)

S5 File DataSource. Source database.
(XLSX)

S6 File DataManagement-commands. Syntax of all management we have done on DataSource.xlsx data.
(TXT)

S7 File DataManagenent-output. List of the results of data management and analyses.
(XLSX)
