# Supplementary material for: Clomiphene citrate effect in obese men with low serum testosterone treated with metformin due to dysmetabolic disorders: A randomized, double-blind, placebo-controlled study
Source: PLoS One. 2017 Sep 8;12(9):e0183369. doi: 10.1371/journal.pone.0183369 (PMC5590732; doi:10.1371/journal.pone.0183369)
Supplement: S1 File — Original (Italian) version of the protocol. (PDF) [file pone.0183369.s001.pdf]

Codice dello studio UOE/01-2011

Titolo dello studio spontaneo interventistico con farmaco *Effetto della terapia con metformina e clomifene in maschi obesi ipogonadici con o senza diabete di tipo 2 sui livelli plasmatici del testosterone e sui parametri metabolici*

Autori:	Renato Pasquali, Vito A. Giagulli
Fase:	*3*
Documento	*Protocollo UOE/01-2011*
Versione:	*finale*
Data di rilascio:	*26-01-2011*
Numero di pagine:	29

PAGINA DELLE FIRME DEL PROTOCOLLO *(Firme dello Sponsor - proponente dello studio spontaneo)*

Codice del protocollo: UOE/01-2011

Renato Pasquali	[illegible]<br>firma	16/2/11<br>data
Vito A. Giagulli	Vito Angelo Giagulli<br>firma	16/2/11<br>data
Antonio Maria Morselli Labate (Statistico)	A M Morselli Labate<br>firma	16/2/11<br>data

DICHIARAZIONE DELLO SPERIMENTATORE:

Codice del protocollo: UOE/01-2011

Dichiaro di aver letto il protocollo ed acconsento a condurre questo studio clinico in accordo a tutti i requisiti del protocollo e secondo le Linee Guida di Buona Pratica Clinica ed i principi della Dichiarazione di Helsinki.

Renato Pasquali ________ firma ________ 16/2/2011 data

INDICE DEI CONTENUTI

1. Introduzione ... 5
2. Obiettivi dello studio ... 6
3. Piano dello studio ... 7
    3.1 Disegno dello studio ... 7
    3.2 Popolazione dello studio ... 8
    3.3 Trattamenti ... 9
    3.4. Visite e valutazioni ... 11
    3.5. Valutazioni di efficacia ... 14
    3.6. Valutazioni di sicurezza ... 14
    3.7 Sottostudio di genetica ... 16
4. Gestione dei dati ed analisi statistica ... 17
    4.1. Gestione dei dati ... 17
    4.2 Metodi statistici ... 18
        - Caratteristiche della casistica, trattamenti e malattie concomitanti ... 18
        - Valutazione di efficacia: Variabile primaria ... 19
        - Valutazione di efficacia: Variabili secondarie ... 19
        - Metodi statistici (valutazione di efficacia) ... 19
        - Valutazione di sicurezza ... 19
        - Dimensione del campione ... 20
5. Procedure amministrative ... 21
6. Bibliografia ... 26

1. Introduzione

Numerosi autori hanno recentemente riportato che i maschi obesi (1, 2) e affetti da diabete mellito tipo 2 (T2DM) o con la sindrome metabolica (SM) (3, 4, 5, 6), possono presentare una riduzione dei livelli plasmatici di testosterone rispetto ai soggetti non affetti da tali disordini metabolici. Inoltre è noto che la prevalenza di obesità e diabete è maggiore nei soggetti in età adulta, e che l'invecchiamento fisiologico nel maschio si caratterizza per un lento e progressivo declino della funzione testicolare, rivelato da una riduzione dei livelli plasmatici di testosterone totale (T) e libero (FT) (7). Pertanto è stato ipotizzato che nel maschio la riduzione dei livelli circolanti di (F)T possa avere un ruolo importante nell'invecchiamento fisiologico, nella genesi dell'insulino-resistenza e del rischio di eventi cardiovascolari che ne consegue (7, 8, 9). Inoltre, è stato dimostrato che il T nel maschio può modulare positivamente la insulino-sensibilità e la secrezione incretinica indipendentemente da variazioni della composizione corporea, come recentemente è stato dimostrato in un gruppo di soggetti normali sottoposti a "clamp iperinsulinemico euglicemico" e a variazione della testosteronemia e degli estrogeni sierici grazie alla somministrazione di un inibitore dell'aromatasi (Letrozolo) (10).

E' generalmente accettato che nel maschio l'estradiolo periferico è il principale fattore che regola la secrezione ipotalamo-ipofisaria di gonadotropine, in particolare di LH, e conseguentemente dei livelli plasmatici del (F)T (11,12). Tuttavia nell'invecchiamento maschile la riduzione del (F)T non è accompagnata da una riduzione consensuale del 17 β estradiolo (E2), che può persino essere aumentata nel maschio obeso (2).

Recenti studi hanno dimostrato l'efficacia del trattamento con gli antiestrogeni e, in particolare, del Clomifene Citrato (CC) nell'aumentare i livelli plasmatici del T nei maschi anziani (14,15) in soggetti obesi ed ipogonadici (13) o nel migliorare la spermatogenesi in maschi infertili (16-19). Il CC blocca il feedback inibitorio dell'estradiolo a livello ipotalamico, aumentando così il rilascio ipofisario di gonadotropine, LH e FSH, stimolano rispettivamente le cellule di Leydig e del Sertoli aumentando i livelli plasmatici di testosterone e favorendo la spermatogenesi (20).

Pertanto l'obiettivo dello studio sarà valutare gli effetti della terapia con clomifene citrato (**25 mg/die - Serofene**) (CC) e metformina (**2 g/die - Glucophage 1000**) (MET), farmaco utilizzato per il trattamento dei pazienti obesi con T2DM o alterata tolleranza glucidica, per la documentata efficacia nel migliorare la sensibilità insulinica (21), sui livelli plasmatici di T in soggetti obesi, di età compresa tra 35 e 55 anni, con ipogondismo e alterata tolleranza glucidica (IGT) o diabete mellito conclamato. Inoltre verrà studiato l'effetto dell'atteso incremento del T sui parametri metabolici confrontando il trattamento con CC + MET rispetto al trattamento con sola MET in tutti i soggetti reclutati nello studio e suddivisi in funzione dello stato di tolleranza glucidica.
Tale studio potrà mettere in evidenza il ruolo del T nel migliorare il quadro metabolico nei soggetti ipogonadici obesi con alterata tolleranza glucidica o diabete, aprendo nuove prospettive terapeutiche.

2. Obiettivi dello studio

Obiettivo primario dello studio:
- valutare l'effetto della terapia con MET e CC sui livelli plasmatici di testosterone.

Obiettivi secondari dello studio:
- effetto della terapia con MET e CC su:

1) livelli plasmatici di glicemia, HbA1c, insulina e C-peptide, e indici di insulino-resistenza (HOMA-IR, Homeostasis model assessment - insulin resistance)

2) altri parametri metabolici (acidi grassi liberi (FFA), colesterolo totale, HDL, LDL, trigliceridi);

3) livelli circolanti dei markers di infiammazione cronica (fibrinogeno, PCR) e delle di citochine infiammatorie (TNF-α, IL-6);

4) livelli plasmatici degli ormoni secreti dal tessuto adiposo (adiponectina, leptina);

5) livelli plasmatici degli endocannabinoidi.

3. Piano dello studio

3.1 Disegno dello studio

Lo studio è randomizzato, in doppio cieco, controllato con placebo, in cross-over, multicentrico in una popolazione di pazienti obesi. Lo studio, della durata complessiva di 30 settimane, si articola in una fase di selezione della popolazione ed una fase di trattamento in cross-over con un periodo di wash-out.

I soggetti, stratificati per la presenza di diabete mellito (T2DM) o alterata tolleranza al glucosio (IGT), verranno randomizzati in due gruppi per l'assegnazione al trattamento farmacologico con metformina (MET), alla dose di 2000 mg al giorno, associata a clomifene (CC) alla dose di 25 mg al giorno (Trattamento A), oppure metformina (MET) alla dose di 2000 mg al giorno più placebo (PLAC) (Trattamento B).

Il disegno in cross-over prevede una prima fase di assegnazione ad un trattamento (A o B) per 12 settimane, seguita da un periodo di wash-out farmacologico di 6 settimane al termine delle quali i soggetti verrano assegnati all'altro trattamento (B o A) (vedi schema 1).

Tutti i pazienti arruolati osserveranno un regime alimentare ipocalorico (1600 kcal/die) associato a moderata attività fisica (25-30 minuti di cammino al giorno) come raccomandato dall'American Diabetes Association (25).

Schema 1

**Fase**	Pre-randomizzazione		Trattamento in doppio cieco				
**Periodo**	Selezione	Basale	Trattamento		Wash-out	Trattamento	
**Settimana**	-1	0	6	12	12-18	24	30
**Visita**	V0	V1	V2	V3	V4	V5	V6
**Trattamento**	Nessuno	Nessuno	MET + CC (A)<br>Oppure<br>MET + plac (B)			MET + CC (A)<br>Oppure<br>MET + plac (B)	

3.2 Popolazione dello studio

La popolazione in studio sarà costituita da 24 pazienti ambulatoriali obesi, di cui 12 affetti da diabete mellito di tipo 2 e 12 con alterata tolleranza glucidica (IGT). Nessun soggetto dovrà essere in terapia farmacologica per il diabete o per altre alterazioni metaboliche definite nella sindrome metabolica (pazienti *naif*).

Criteri di inclusione

I soggetti selezionati dovranno soddisfare i seguenti criteri:

- maschi di eta compresa tra 35 e 55 anni
- obesità, definita in accordo con i criteri della WHO per un indice di massa corporea (BMI) > 30 kg/m^2
- diagnosi di diabete o di alterata tolleranza glucidica secondo i criteri definiti dall'American Diabetes Association (ADA) nel 2009 (25): in particolare saranno considerati affetti da alterata tolleranza glucidica i soggetti con glicemia alla seconda ora dopo carico orale di glocosio (OGTT) compresa tra 140 e 199 mg/dl, e affetti da diabete i soggetti con glicemia a digiuno > 126 mg/dl o con glicemia alla seconda ora dopo carico orale di glocosio (OGTT) > 200 mg/dl
- HbA1c < 8.5%
- sindrome metabolica, definita secondo le linee guida ATP III, ovvero per la presenza contemporanea di 3 o più dei seguenti disordini: obesità centrale (circonferenza vita ≥ 102 cm); ipertensione arteriosa (PAS ≥ 130 mmHg o PAD ≥ 85 mmHg o terapia farmacologica per ipertensione); ipertrigliceridemia (≥ 150 mg/dL o terapia farmacologica ipertrigliceridemia); ridotto colesterolo HDL (< 40 mg/dL o terapia farmacologica per basso colesterolo HDL) (26)
  - ipogonadismo, definito per livelli circolanti di testosterone totale ≤ 3 ng/ml (7)
  - firma del consenso informato.

Criteri di esclusione

- Pazienti con ipogonadismo di natura primitiva o secondaria associato a malattie genetiche o a processi infiltrativi o distruttivi a carico degli organi endocrini (testicolo, ipotalamo-ipofisi)

- Pazienti in terapia farmacologica con ipoglicemizzanti orali in atto o nei 3 mesi precedenti l'inizio dello studio
- Pazienti in terapia farmacologica con ipolipemizzanti in atto o nei 3 mesi precedenti l'inizio dello studio
- Nota o sospetta ipersensibilità al farmaco od alla classe farmacologica in studio;
- Pazienti con gravi condizioni cliniche che, a giudizio dello sperimentatore, controindicano la partecipazione del paziente allo studio;
- Utilizzo di farmaci sperimentali per via sistemica negli ultimi 3 mesi prima dell'inclusione nello studio.
- Pazienti non in grado di seguire le procedure previste dal protocollo.

3.3 Trattamenti

Trattamenti in studio

La metformina verrà fornita come compresse da 1000 mg sotto forma delle confezioni già disponibili in commercio. La metformina verrà somministrata al dosaggio di 1000 mg, 2 volte al giorno da assumere durante o dopo i pasti principali.

Il clomifene, fornito in compresse di Serofene da 50 mg dalla ditta produttrice Merck Serono verrà allestito come capsule da 25 mg dal Laboratorio galenico della Farmacia Ospedaliera del Policlinico di Bologna.

Il clomifene verrà somministrato al dosaggio di 25 mg, una volta al giorno. Il placebo verrà allestito dalla Famacia Ospedaliera nella stessa forma farmaceutica del clomifene, e somministrato una volta al giorno.

Assegnazione del trattamento

Confermata l'eligibilità, un unico codice identificativo verrà assegnato dallo Sperimentatore ad ogni singolo centro sperimentale ai paziente in studio. Il codice è costituito dal numero del centro seguito dal numero progressivo di entrata nello studio presso il centro sperimentale. Una volta assegnati, i codici dei pazienti che per qualsiasi motivo non continuano lo studio non potranno più essere riutilizzati

I pazienti che soddisfano i criteri di inclusione e di esclusione verranno randomizzati ad uno dei trattamenti in studio (trattamento A vs trattamento B).

L'assegnazione del paziente ad uno dei gruppi di trattamento avviene secondo la lista di randomizzazione generata attraverso computer da Investigational Drug Service (IDS) presso la Farmacia Ospedaliera del Centro Coordinatore rispettando i criteri di doppia cecità.

La lista di randomizzazione verrà inoltrata, nella parte di loro competenza, ai farmacisti ospedalieri individuati come riferimento per lo studio in ogni centro sperimentale e non potrà essere divulgata dutrante il corso della sperimentazione ad altri sperimentatori coinvolti nello studio clinico in oggetto. In seguito alla richiesta di randomizzazione, inoltrata dagli sperimentatori al proprio farmacista referente, la farmacia ospedaliera di ogni centro assegna il numero random, prepara il farmaco sperimentale/placebo e lo invia allo sperimentatore richiedente garantendo la doppia cecità.

Al paziente dovrà essere assegnato il codice di randomizzazione piu' basso disponibile.

Se durante lo studio si rendesse necessario conoscere il codice di trattamento di un paziente, si veda nel Cap. 5 (procedure amministrative) la procedura per l'apertura dei codici di emergenza. L'apertura dei codici di emergenza è consentita in casi eccezionali unicamente per intraprendere adeguate misure terapeutiche altrimenti non identificabili.

Terapie concomitanti

Non sarà ammesso durante lo studio l'uso dei seguenti farmaci:

- farmaci ipoglicemizzanti orali diversi dalla metformina e altri agenti antidiabetici
- glucocorticoidi di sintesi per via sistemica
- terapia ormonale (androgeni, antiandrogeni)
- farmaci ipolipemizzanti.

Sarà consentito durante lo studio l'utilizzo di:

- farmaci ipolipemizzanti in caso di valori di LDL-colesterolo non in target dopo 6 mesi dall'inizio dello studio (LDL >100 mg/dl)
- farmaci anti-ipertensivi.

Interruzione del trattamento

Ogni paziente ha piena facoltà di interrompere la sua partecipazione allo studio in qualsiasi momento; inoltre, qualora si ritenga che sia di beneficio alla sua salute, la partecipazione del paziente allo studio potrà essere interrotta. In particolare in caso di necessità di aggiungere in terapia un secondo farmaco ipoglicemizzante orale per inadeguato controllo metabolico (HbA1c $\geq$ 8.5 %) verrà interrotta la partecipazione del paziente allo studio.

Aderenza al trattamento

Ai pazienti verrà chiesto di consegnare ad ogni visita ed al termine dello studio tutti i farmaci in studio non utilizzati. La quantità di farmaci riconsegnata dovrà essere documentata. Lo sperimentatore verificherà l'aderenza del paziente alla posologia prescritta ed agirà opportunamente in caso di non osservanza.

In caso di interruzione del trattamento sperimentale di durata superiore a 7 giorni consecutivi il paziente verrà escluso dallo studio e non potrà proseguire con il trattamento sperimentale (drop-out).

3.4. Visite e valutazioni

Visita di selezione

Prima di iniziare ogni procedura specifica dello studio, il ricercatore dovrà ottenere dal paziente il consenso informato scritto firmato. Le valutazioni di screening comprendono esami di laboratorio ematochimici e ormonali, un test da carico orale di glucosio ed una visita clinica. Solo per i pazienti eleggibili le valutazioni verranno riportate in CRF in corrispondenza della visita V0.

Schema delle visite e delle valutazioni

Il numero di visite complessivo è 7 (V0-V6, secondo schema delle valutazioni). Le procedure relative alle singole visite sono riportate nello schema delle valutazioni. Le visite potranno essere anticipate/posticipate di 7 giorni.

Schema delle valutazioni

Numero della visita	V0	V1	V2	V3		V4	V5	V6
Trattamento	Selezione	Randomizzazione (assegnazione A o B)	A o B	A o B	Wash-out	Cross-over (assegnazione B o A)	B o A	B o A
Periodo	giorni – 7/0	sett. 0 ±7 gg	sett. 6 ±7 gg	sett. 12 ±7 gg		sett. 18 ±7 gg	sett. 24 ±7 gg	sett. 30 ±7 gg
Consenso informato	X							
Storia clinica<br>Terapie concomitanti in passato	X<br>X	X<br>X				X<br>X		
Terapie concomitanti in atto	X	X	X	X		X	X	X
Obiettività generale<br>Questionari qADAM, CDQ, CSD, Androtest, IIEF		X<br>X	X	X<br>X		X<br>X	X	X<br>X
Esami di laboratorio	X			X		X		X
Visita dietista		X				X		
Criteri di incl./escl.	X	X						
Consegna del farmaco in studio		X				X		
Eventi avversi		X	X	X		X	X	X
Valutazione del grado di soddisfazione per il trattamento				X				X

A: trattamento A (CC+MET); B: trattamento B (PLAC+MET)

Esami di laboratorio

Allo screening verranno effettuati gli esami ematochimici ed ormonali in condizioni di digiuno. In particolare allo screening tutti i soggetti effettueranno un prelievo di sangue basale per determinazione di:

- parametri metabolici (glicemia, insulinemia, c-peptide, HbA1c, acidi grassi liberi (FFA), colesterolo totale, HDL, LDL, trigliceridi, GOT, GPT, gGT, fosfatasi alcalina, protide mia totale e frazionata, creatinina);

- markers di infiammazione cronica (fibrinogeno, PCR) e citochine infiammatorie (TNF-α, IL-6);
- omoni e proteine (testosterone totale ed SHBG, TSH, fT4);
- test da carico orale di glucosio (OGTT) con 75 g di glucosio con dosaggio di glicemia, insulinemia e C-peptide (tempi 0', 30'. 60', 90', 120').

Verranno inoltre prelevate aliqote di sangue e siero per il dosaggio di ormoni secreti dal tessuto adiposo (adiponectina, leptina) ed endocannabinoidi.

Gli esami sopraelencati verranno effettuati al tempo della visita V0, V3, V4 e V6.

L'OGTT verrà effettuato solo a V0 per definire lo stato di tolleranza glucidica ed inclusione nello studio.

Le aliquote di siero e di plasma e sangue intero di ciascun soggetto saranno conservate in congelatore a -80°C fino alla determinazione analitica; le analisi sui campioni di tutti i soggetti verranno effettuate al termine dello studio per evitare variazioni intra-assay. Saranno anche determinati i livelli di testosterone con metodo HPLC-MS/MS (high-performance liquid chromatography with tandem mass spectrometry) (27), mentre il FT sarà calcolato utilizzando la formula di Vermeulen (28). I livelli plasmatici di SHBG saranno misurati con metodo immunometrico, insulina e C-peptide vennaro misurati con metodo ECLIA, PCR con metodo Turbidimetrico/immunonefel., colesterolo totale con metodo CHOD-PAP, HDL con metodo enzimatico colorimetrico omogeneo, trigliceridi con metodo GPO-PAP, FFA con metodo ACS ACOD, IL-6 con chemioluminescenza, e TNF-α immunoenzimatico. Come indice di insulino-resistenza verrà effettuato il calcolo dell'HOMA-IR secondo Matthews (29). Per il dosaggio di adiponectina e leptina verrà utilizzato il metodo RIA (kit per leptina: Millipore HL-81HK, per adiponectina Millipore HADP-61HK). Per la determinazione degli endocannabinoidi verrà utilizzato il metodo HPLC-MS/MS (30).

Le determinazioni verranno effettuate presso il Laboratorio Centralizzatro del Policlinico S. Orsola-Malpighi e presso il Centro di Ricerca di Biologia Applicata dell'Università di Bologna.

3.5. Valutazioni di efficacia

L' obiettivo primario dello studio è valutare l'effetto della terapia con metformina e con clomifene sui livelli plasmatici di testosterone.

3.6. Valutazioni di sicurezza

La valutazione della sicurezza del farmaco consisterà nel monitoraggio e nella registrazione degli eventi avversi, degli eventi avversi seri, degli esami di laboratorio e nella misurazione dei segni vitali.

Eventi avversi

Le informazioni relative a tutti gli eventi avversi, sia quelli riferiti spontaneamente dal soggetto sia quelli riscontrati dallo Sperimentatore a seguito di domande specifiche che quelli evidenziati dall'esame fisico del paziente, da indagini di laboratorio od altro, verranno raccolti, registrati sulla CRF e seguiti come appropriato.

Si definisce **evento avverso** ogni segno, sintomo o condizione clinica indesiderati che si verifichi dopo l'inizio del trattamento con il farmaco (o terapia) somministrato, anche se non c'è relazione di causalità tra l'evento ed il farmaco (o terapia) somministrato. Il farmaco (o terapia) somministrato include il farmaco (o terapia) oggetto di valutazione ed ogni farmaco (o terapia) di confronto o placebo, somministrati durante una qualunque fase dello studio.

Le condizioni cliniche/malattie presenti già prima dell'inizio del trattamento con il farmaco somministrato sono considerate eventi avversi solo se peggiorano dopo l'inizio del trattamento con il farmaco somministrato. Eventuali anomalie evidenziate dagli esami di laboratorio o da test costituiscono eventi avversi solo se causano segni o sintomi, se sono considerate rilevanti da un punto di vista clinico o se richiedono una terapia, e vengono registrate nella CRF di seguito ai segni, sintomi od alla diagnosi associate.

Per quanto possibile, ogni evento avverso verrà descritto in termini di livello di severità (lieve, moderato, grave) (livelli 1 - 3).

Eventi Avversi Seri

Le informazioni relative a tutti gli eventi avversi seri verranno raccolte e registrate sul modulo di segnalazione degli Eventi Avversi Seri. Per garantire la sicurezza del paziente tutti gli eventi avversi seri devono essere segnalati entro 24 ore da quando lo Sperimentatore ne viene a conoscenza.

Sono definiti **Eventi Avversi Seri** quegli eventi avversi che:

- risultano fatali;
- pongono il paziente in pericolo di vita;
- richiedono il ricovero del paziente o ne prolungano la degenza;
- comportano un'invalidità/incapacità significativa e persistente anche se non necessariamente permanente;
- sono significative dal punto di vista medico nel senso che possono danneggiare il paziente e richiedono un intervento medico o chirurgico per prevenire le situazioni sopra elencate.

Non sono considerati eventi avversi seri eventuali ospedalizzazioni per:

- trattamenti di routine o monitoraggio della condizione clinica oggetto di osservazione, non associati ad un peggioramento della condizione clinica stessa;
- trattamenti di elezione o programmati in precedenza per condizioni cliniche preesistenti che non sono correlate alla patologia oggetto di osservazione e che non hanno subito un peggioramento;
- cure generali (in ospedale od altri istituti) non associate ad alcun peggioramento delle condizioni cliniche generali;
- trattamenti di emergenza a livello ambulatoriale per eventi che **non** soddisfano la definizione di eventi avversi seri e che **non** comportano l'ingresso in ospedale.

Deve essere riportato anche ogni evento avverso serio che si verifica dopo che il paziente ha fornito il consenso informato e fino a 4 settimane dopo l'ultima somministrazione del farmaco in studio. Gli eventi avversi seri che si verificano dopo che sono trascorse 4 settimane da quando il paziente ha assunto l'ultima dose del farmaco in studio, devono essere riportati solo se si sospetta una relazione di causalità con il farmaco (o terapia) somministrati durante lo studio.

Lo Sperimentatore deve compilare il modulo di segnalazione degli eventi avversi seri, valutare la relazione di causalità con il farmaco in studio.

Qualsiasi episodio ricorrente, complicazione o progressione di un evento già segnalato deve essere comunicato come follow-up di quell'evento.

Le informazioni di follow-up devono essere comunicate con una nuova scheda, specificando che si tratta di follow-up di un evento già segnalato ed indicando la data della avvenuta notifica iniziale.

Persone di riferimento

I numeri di telefono e fax delle persone di riferimento per la segnalazione degli Eventi Avversi Seri sono riportati nell'Investigator Folder.

3.7 Sottostudio di genetica

Le concentrazioni plasmatiche di testosterone totale (T) e della sua quota libera (FT) presentano una considerevole variabilità interindividuale. Esistono fattori ambientali (obesità, fumo, ecc) (21) e fattori genetici (22) in grado di influenzare i livelli circolanti di testosterone. Studi su ampie popolazioni di soggetti maschi sani hanno dimostrato che la variabilità interindividuale nelle concentrazioni di FT riflette differenze nella sensibilità androgenica. In particolare è stata documentata una correlazione diretta tra le concentrazioni del FT e la lunghezza delle triplette CAG nell'esone 1 del gene del recettore degli androgeni (AR) codificante un tratto glutamminico polimorfico (23,24).

Pertanto il polimorfismo CAG del recettore degli androgeni in soggetti obesi con alterata tolleranza al glucosio o con diabete conclamato potrebbe spiegare le differenze interindividuali nella risposta terapeutica, in particolare sul quadro metabolico, nonostante l'atteso aumento delle concentrazioni di testosterone.

A tutti i soggetti che partecipano allo studio verrà proposta la partecipazione al sottostudio di genetica che prevede la determinazione del polimorfismo CAG del recettore degli androgeni.

Il polimorfismo CAG del primo esone del recettore degli androgeni verrà misurato con metodo già descritto in letteratura (31) presso il Centro di Genetica e Biologia Molecolare del

centro di Ricerche cliniche ed Ormonali "Telesforo" (via Rosati, 137/C, Foggia , responsabile del Laboratorio Dr. Domenico Carbone). Da un campione di 300 mcl di samgue verrà estratto il DNA (AB Analitica srl, Advance Biomedicine, REF. 05-42, Padova, Italia) che verrà amplificato con appositi primers con il metodo della polimerasi (PCR). La conta delle triplette CAG sarà effettuata tramite una analizzatore automatico [ABI PRISM 3100 Genetic Analyzer (Perkim-Elmer Cop.)]. La determinazione della lunghezza delle triplette CAG (260-320 bp) verrà effettuato due volte in due separate determinazioni.

4. Gestione dei dati ed analisi statistica

4.1. Gestione dei dati

Il personale designato dallo Sperimentatore dovrà riportare le informazioni richieste dal protocollo sulla Scheda Raccolta Dati (CRF).

I dati della CRF verranno inseriti centralmente da personale designato dallo Sperimentatore mediante singolo data entry con verifica elettronica dei dati. Gli elementi di testo (es. commenti) verranno verificati manualmente. I dati inseriti verranno successivamente controllati mediante programmi di validazione e controllo di listati. Gli errori ovvi verranno corretti direttamente dal personale di data management, altri errori od omissioni verranno verificate sulle cartelle cliniche.

Le informazioni relative ai trattamenti concomitanti verranno codificate mediante la WHO Drug Reference List, che usa il sistema di classificazione Anatomic Therapeutic Chemical (ATC). Le patologie concomitanti e gli eventi avversi verranno codificati usando la terminologia della classificazione ICD9.

Il database verrà chiuso una volta dichiarato completo ed accurato. Qualsiasi modifica ai dati successiva alla chiusura potrà unicamente essere effettuata con l'accordo scritto del responsabile clinico dello studio spontaneo.

4.2 Metodi statistici

L'obiettivo di questo studio è valutare l'efficacia del trattamento con MET e CC nell'aumentare i livelli di testosterone in soggetti obesi con IGT o T2DM in confronto al trattamento con MET e PLAC.

I dati raccolti verranno raggruppati e riassunti rispetto alle variabili demografiche, alle caratteristiche basali ed alle valutazioni di efficacia e sicurezza.

Le analisi esploratorie saranno effettuate utilizzando statistiche descrittive:

- medie, deviazioni standard, frequenze assolute e relative, range, intervalli di confidenza.

I dati saranno presentati sia per la popolazione intent-to-treat (ossia tutti i pazienti che hanno assunto almeno una dose del farmaco di studio) che per la popolazione per-protocol (ossia tutti i pazienti che hanno completato lo studio senza violazioni maggiori del protocollo).

Le valutazioni di sicurezza saranno basate principalmente sulla frequenza degli eventi avversi, includendo tutti gli eventi avversi seri. Gli eventi avversi verranno riassunti presentando per ogni gruppo di trattamento il numero e la percentuale di pazienti che hanno avuto un qualsiasi evento avverso, un evento avverso in uno specifico apparato dell'organismo ed uno specifico evento avverso. Ogni altra informazione raccolta (i.e. la gravità o la relazione con il farmaco di studio) verrà codificata come appropriato.

Verranno inoltre prodotte liste analitiche che riportano informazioni dettagliate relativamente a:

- pazienti che hanno interrotto lo studio e relativi motivi;
- pazienti che hanno interrotto lo studio per eventi avversi;
- pazienti che hanno avuto eventi avversi seri;
- pazienti con valori di esami di laboratorio fuori dai range predefiniti.

Caratteristiche della casistica, trattamenti e malattie concomitanti

I dati relativi alle caratteristiche demografiche e basali ed alle osservazioni e misure di efficacia e sicurezza verranno opportunamente riassunti. Le caratteristiche di somministrazione del farmaco in studio e degli altri trattamenti concomitanti verranno riassunte, analogamente alle principali malattie concomitanti registrate all'ammissione in

studio. Verranno inoltre listati i pazienti che hanno interrotto lo studio e descritti analiticamente i relativi motivi.

Le analisi esploratorie saranno effettuate utilizzando statistiche descrittive suddividendo i pazienti per tipo di trattamento, successione del trattamento e tolleranza glucidica.

Verranno riportate: medie, deviazioni standard (DS), frequenze assolute e relative, range, intervalli di confidenza.

Valutazione di efficacia: Variabile primaria

La popolazione su cui verrà effettuata l'analisi primaria di efficacia e' costituita da tutti i pazienti randomizzati che abbiano assunto entrambi i trattamenti.

La variabile primaria di efficacia è l'incremento dei livelli plasmatici di testosterone dopo trattamento.

Valutazione di efficacia: Variabili secondarie

Le variabili secondarie di efficacia sono un miglioramento del controllo metabolico, definiti da riduzione dei livelli di glicemia e HbA1c, insulinemia e C-peptide, riduzione degli indici di insulino-resistenza (HOMA-IR), miglioramento dell'assetto lipidico (riduzione dei livelli di colesterolo totale, LDL e trigliceridi, e aumento dei livelli di HDL, riduzione dei livelli di FFA), riduzione degli indici di infiammazione cronica (fibrinogeno, PCR) e delle citochine infiammatorie (TNF-α, IL-6) dopo trattamento.

Metodi statistici (Valutazione di efficacia)

La normalità dei dati verrà verificata utilizzando ol test di Kolmogorov-Smirnov.

I dati verranno valutati mediante l'analisi della varianza (ANOVA) per misure ripetute a tre vie (tipo di trattamento, successione del trattamento, tolleranza glucidica).

Valutazione di sicurezza

La popolazione su cui verrà effettuata l'analisi di sicurezza e' la 'Popolazione di sicurezza' costituita da tutti i pazienti inclusi nello studio che abbiano assunto almeno una dose del farmaco in studio.

Gli eventi avversi verranno riassunti presentando il numero e la percentuale di pazienti con ogni tipologia di evento, classificata per body system e per preferred term. Le altre informazioni raccolte (es. gravità, relazione causale) verranno listate così come i pazienti con eventi avversi seri.

Verranno inoltre listati i pazienti che hanno avuto eventi avversi seri e che hanno interrotto lo studio per eventi avversi.

I valori degli esami di laboratorio verranno riassunti in opportune tabelle che riporteranno la frequenza dei valori al di fuori di un intervallo predeterminato. I valori che rappresentano anormalità rilevanti verranno listati.

Gli altri dati di sicurezza (es. segni vitali ed esami specifici) verranno valutati in modo appropriato.

Dimensione del campione

Lo studio e' stato disegnato per dimostrare che l'effetto dell'associazione metformina + clomifene e' superiore all'effetto della sola metformina sui livelli di testosterone dopo 12 settimane di trattamento.

E' stato calcolato che per avere una probabilità di almeno 80% di trovare, con un livello di significatività del 5% (due code), una differenza tra i trattamenti del livello di testosterone di 2,8 ng/mL con DS di 1.5 ng/mL, deve essere valutato un totale di 20 pazienti (28,29). Pertanto si è deciso di randomizzare 24 pazienti in modo da poter compensare una frequenza di drop-out dell'ordine del 10-20%.

La dimensione del campione è stata calcolata con il programma "PS Power and Sample Size Calculations" del Dipartimento di Statistica della Vanderbilt University, Nashville, TN, USA (Version 3.0.12; http://biostat.mc.vanderbilt.edu/twiki/bin/view/Main/PowerSampleSize) che utilizza le procedure di Dupont and Plummer (28, 29).

5. Procedure amministrative

Norme di Buona Pratica Clinica

Questo studio verrà condotto in accordo ai principi della Good Clinical Practice (30), alla dichiarazione di Helsinki ed alle normative nazionali in materia di conduzione delle sperimentazioni cliniche. Lo sperimentatore, firmando il protocollo, acconsente ad aderire alle procedure ed alle istruzioni in esso contenute ed a svolgere lo studio secondo GCP, la Dichiarazione di Helsinki e le normative nazionali che disciplinano le sperimentazioni cliniche.

Emendamenti al protocollo o ogni altra modifica alla conduzione dello studio

Qualunque modifica al protocollo sarà apportata sotto forma di emendamento. Non sono permesse modifiche al protocollo durante il periodo di studio. Ogni modifica imprevista nella conduzione dello studio sarà registrata nel "Clinical Study Report".

Comitati etici e consenso informato

Il protocollo di studio, ogni emendamento del protocollo, il consenso informato ed ogni altra informazione per i pazienti dovranno essere approvati dal Comitato Etico della struttura ove opera lo Sperimentatore.

Per quanto concerne gli emendamenti, lo Sperimentatore può subito applicarli previa comunicazione scritta al Comitato Etico, senza aspettare l'approvazione del Comitato Etico, qualora sia in gioco la sicurezza dei pazienti partecipanti allo studio. Inoltre, se lo Sperimentatore ritiene che per ragioni di sicurezza dei pazienti sia necessario apportare immediatamente una modifica al protocollo, deve metterne a conoscenza il Comitato Etico del centro entro 10 giorni lavorativi.

Per partecipare allo studio ogni paziente dovrà fornire il consenso informato scritto (vedi anche paragrafo 3.2 - Popolazione dello studio).

Archivio della documentazione

Lo Sperimentatore è responsabile dell'archiviazione e conservazione dei documenti essenziali dello studio, prima, durante la conduzione e dopo il completamento o l'interruzione dello studio stesso, in accordo a quanto/e per il tempo previsto dalla normativa vigente e dalle GCP.

I dati raccolti sulla CRF saranno in forma rigorosamente anonima ed il soggetto verrà unicamente identificato con un numero e con le iniziali.

Lo Sperimentatore dovrà conservare i dati originali del paziente (ad es. informazioni demografiche e mediche, dati di laboratorio, elettrocardiogrammi etc.) ed una copia del consenso informato scritto firmato. Per alcuni dati può essere stabilito, prima che lo studio inizi, che vengano scritti direttamente sulla CRF, che quindi in questo caso fungerà da dato originale.

Procedure di emergenza per la sospensione della cecità

Contemporaneamente all'invio della lista di randomizzazione, che verrà inoltrata ai farmacisti ospedalieri individuati come riferimento per lo studio in ogni centro sperimentale e che non potrà essere divulgata dutrante il corso della sperimentazione ad altri sperimentatori coinvolti nello studio clinico in oggetto, verrà inoltrata allo Sperimentatore di ogni singolo centro sperimentale le singole buste sigillate corrispondenti alla parte della lista di randomizzazione per specifico centro sperimentale. Ogni busta identificata con il numero di randomizzazione contiene l'informazione sul tipo di trattamento (trattamento farmacologico/placebo) attribuito al paziente. La busta sigilata potrà essere apèerta dallo Sperimentatore di ogni singolo centro solo in caso di emergenza. Lo Speirmantatore dovrà nel contempo documentare la ragione dell'apertura del codice, la data/ora dell'apertura della busta e il documento contenutop nella busta sigillata.

Il ricercatore dovrà inoltre avvisare immediatamente il resposabile dello studio.

Ispezioni/Verifiche

Lo Sperimentatore o il personale delegato potrà condurre delle verifiche durante lo studio per assicurarsi che venga condotto in accordo al protocollo ed alle disposizioni normative applicabili. Anche le Autorità Regolatorie possono condurre ispezioni sullo studio (durante il suo svolgimento oppure dopo che lo studio si è completato). Se un'Autorità Regolatoria richiede un'ispezione, lo Sperimentatore dovrà subito informare il responsabile dello studio. Firmando il protocollo, lo Sperimentatore acconsente sia alle verifiche da parte der responsabile dello studio o del personale delegato che alle ispezioni della Autorità Regolatoria.

Gestione del farmaco di studio

Il farmaco di studio verrà fornito interamente dallo Sperimentatore.

La metformina verrà fornita come compresse da 1000 mg in confezioni già disponibili in commercio.

Il clomifene, fornito dalla ditta produttrice Merck Serono come compresse di Serofene da 25 mg, verrà allestito dalla Farmacia dell'Azienda Ospedaliero-universitaria di Bologna come capsule da 25 mg per la somministrazione al paziente.

Lo Sperimentatore dovrà assicurare che il farmaco in studio sia usato in conformità al protocollo.

Lo Sperimentatore è responsabile della conservazione del farmaco in un luogo sicuro, con accesso limitato. Il farmaco dovrà essere mantenuto in accordo alle condizioni di conservazione riportate sulla confezione.

Procedura di allestimento del campione sperimentale in farmacia

La Farmacia dell'Azienda Ospedaliero-universitaria di Bologna allestisce medicinali sperimentali ai sensi e nel rispetto dei requisiti previsti dall'articolo 15 del DL 06/11/2007 n. 200 in conformità alle Norme di Buona Preparazione dei Medicinali, secondo la vigente edizione della Farmacopea Ufficiale Italiana, per garantire la qualità, l'efficacia e la sicurezza del medicinale allestito.

Il Laboratorio Galenico della Farmacia Ospedaliera del Policlinico di Bologna, allestirà le capsule di placebo (amido di riso) e di clomifene 25 mg.

Metodo di preparazione delle capsule di Clomifene 25 mg o placebo

Si triturano, in apposito mortaio, le compresse di Serofene da 25 mg fino ad ottenere una polvere fine.

La polvere di clomifene così ottenuta ed eventuali eccipienti e per il placebo la polvere di amido di riso si mescolano secondo il metodo delle diluizioni progressive e si rendono omogenei mediante miscelazione. Qualora siano presenti in quantità ridotta può essere conveniente aggiungere un eccipiente diluente inerte per aumentare il volume e consentire di ridurre statisticamente la possibilità di errore durante la suddivisione.

Il medicinale sperimentale o il placebo saranno preparati a lotti con un'opercolatrice e orientatrice automatiche con vassoio da 300 capsule.

La validità del preparato è di 6 mesi a temperatura ambiente con una eventuale possibilità di estenderla ad 1 anno sulla base di specifiche valutazioni da parte del farmacista preparatore.

La preparazione sarà documentata su apposito registro dedicato alle sperimentazioni cliniche sul quale vengono registrati il n. di lotto, la data di preparazione, la composizione quali-quantitativa, la validità, i documenti di scarico dal magazzino farmaci, la firma del farmacista preparatore, l'idoneità alla distribuzione del prodotto finito.

Le capsule di farmaco sperimentale/placebo saranno poi confezionate in appositi contenitori di polietilene ed etichettati.

Inoltre, prima della distribuzione, il Farmacista responsabile eseguirà i seguenti controlli:

- verifica della correttezza delle procedure eseguite
- controllo dell'aspetto e della tenuta delle capsule
- controllo del numero di capsule allestite e della quantità da dispensare
- controllo del confezionamento primario e secondario
- verifica della corretta compilazione dell'etichetta
- saggio uniformità di massa sul lotto.

Pubblicazione dei risultati

Ogni presentazione formale o pubblicazione dei dati derivati da questo studio deve intendersi come una pubblicazione congiunta da parte dello Sperimentatore e responsabile dello studio. Per gli studi multicentrici, è mandatorio che la prima pubblicazione sia basata su i dati di tutti i centri, analizzati secondo protocollo. Gli Sperimentatori che partecipano a studi multicentrici acconsentono a non presentare dati di un singolo centro o di un piccolo gruppo di centri a meno che non vi sia un consenso formale da parte degli altri Sperimentatori e del Dott. VA Giagulli (proponente dello studio spontaneo) che deve ricevere copia di ogni comunicazione che si intende pubblicare in anticipo sulla pubblicazione stessa.

Riservatezza e Confidenzialità

I documenti dello studio forniti dallo Sperimentatore (es. protocollo, CRF ed altro) dovranno essere conservati in luogo sicuro per assicurare il mantenimento della confidenzialità e

riservatezza. Le informazioni ed i documenti forniti dallo Sperimentatopre non potranno essere divulgati ad altri senza autorizzazione scritta del responsabile dello studio, tranne che nella misura necessaria per ottenere il consenso del paziente alla partecipazione allo studio.

Interruzione dello studio

Il responsabile dello studio si riserva la facoltà di interrompere lo studio nel caso di eventi avversi seri associato all'assunzione del trattamento sperimentale in due o più soggetti reclutati.

Persone di riferimento

I numeri di telefono e fax delle persone di riferimento per la conduzione dello studio sono riportati nell'Investigator Folder.

6. Bibliografia

1. Zumoff B, Strain GW, Miller LK, Rosner W, Senie R, Seres DS, Rosenfeld RS. Plasma free and non-sex-hormone-binding-globulin-bound testosterone are decreased in obese men in proportion to their degree of obesity. J Clin Endocrinol Metab. 1990 Oct;71(4):929-31.

2. Giagulli V.A., Kaufman JM, Vermeulen A. Pathogenesis of the decreased androgen levels in obese men. J Clin Endocrinol Metab. 1994 Oct;79(4):997-1000.

3. Pitteloud N, Mootha VK, Dwyer AA, Hardin M, Lee H, Eriksson KF, Tripathy D, Yialamas M, Groop L, Elahi D, Hayes FJ. Relationship between testosterone levels, insulin sensitivity, and mitochondrial function in men. Diabetes Care. 2005 Jul;28(7):1636-42.

4. Ding EL, Song Y, Malik VS, Liu S. Sex differences of endogenous sex hormones and risk of type 2 diabetes: a systematic review and meta-analysis. JAMA. 2006 Mar 15;295(11):1288-99.

5. Chen RY. Wittert GA, Andrews GR. Relative androgen deficiency in relation to obesity and metabolic status in older men. Diabetes Obes Metab. 2006 Jul;8(4):429-35.

6. Grossmann M, Thomas MC, Panagiotopoulos S, Sharpe K, Macisaac RJ, Clarke S, Zajac JD, Jerums G. Low testosterone levels are common and associated with insulin resistance in men with diabetes. J Clin Endocrinol Metab. 2008 May;93(5):1834-40.

7. Kaufman JM & Vermeulen A. The decline of androgen levels in elderly men and its clinical and therapeutic implications. Endocr Rev. 2005 Oct;26(6):833-76.

8. Hak AE, Witteman JC, de Jong FH, Geerlings MI, Hofman A, Pols HA. Low levels of endogenous androgens increase the risk of atherosclerosis in elderly men: the Rotterdam study. J Clin Endocrinol Metab. 2002 Aug;87(8):3632-9.

9. English KM, Mandour O, Steeds RP, Diver MJ, Jones TH, Channer KS. Men with coronary artery disease have lower levels of androgens than men with normal coronary angiograms. Eur Heart J. 2000 Jun;21(11):890-4.

10. Lapauw B, Ouwens M, 't Hart LM, Wuyts B, Holst JJ, T'Sjoen G, Kaufman JM, Ruige JB. Sex steroids affect triglyceride handling, glucose-dependent insulinotropic polypeptide, and insulin sensitivity: a 1-week randomized clinical trial in healthy young men. Diabetes Care. 2010 Aug;33(8):1831-3.

11. T'Sjoen GG, Giagulli VA, Delva H, Crabbe P, De Bacquer D, Kaufman JM. Comparative assessment in young and elderly men of the gonadotropin response to aromatase inhibition. J Clin Endocrinol Metab. 2005 Oct;90(10):5717-22.

12. Raven G, de Jong FH, Kaufman JM, de Ronde W. In men, peripheral estradiol levels directly reflect the action of estrogens at the hypothalamo-pituitary level to inhibit gonadotropin secretion. J Clin Endocrinol Metab. 2006 Sep;91(9):3324-8.

13. Loves S, Ruinemans-Koerts J, de Boer H. Letrozole once a week normalizes serum testosterone in obesity-related male hypogonadism. Eur J Endocrinol. 2008 May;158(5):741-7.

14. Leder BZ, Rohrer JL, Rubin SD, Gallo J, Longcope C. Effects of aromatase inhibition in elderly men with low or borderline-low serum testosterone levels. J Clin Endocrinol Metab. 2004 Mar;89(3):1174-80.

15. Veldhuis JD, Iranmanesh A. Short-term aromatase-enzyme blockade unmasks impaired feedback adaptations in luteinizing hormone and testosterone secretion in older men. J Clin Endocrinol Metab. 2005 Jan;90(1):211-8.

16. Duschek EJ, Gooren LJ, Netelenbos C. Comparison of effects of the rise in serum testosterone by raloxifene and oral testosterone on serum insulin-like growth factor-1 and insulin-like growth factor binding protein-3. Maturitas. 2005 Jul 16;51(3):286-93.

17. Vandekerckhove P, Lilford R, Vail A, Hughes E. WITHDRAWN: Androgens versus placebo or no treatment for idiopathic oligo/asthenospermia. Cochrane Database Syst Rev. 2007 Jul 18;(4):CD000150. Review.

18. Ghanem H, Shamloul R. An evidence-based perspective to the medical treatment of male infertility: a short review. Urol Int. 2009;82(2):125-9.

19. Kaminetsky J, Hemani ML. Clomiphene citrate and enclomiphene for the treatment of hypogonadal androgen deficiency. Expert Opin Investig Drugs. 2009 Dec;18(12):1947-55.

20. Goldstein SR, Siddhanti S, Ciaccia AV, Plouffe L Jr. A pharmacological review of selective oestrogen receptor modulators. Hum Reprod Update. 2000 May-Jun;6(3):212-24.

21. Kaufman JM, Vermeulen A. The decline of androgen levels in elderly men and its clinical and therapeutic implications. Endocr Rev. 2005, 26, 833-876.

22. Vanbillemont G, Lapauw B, Bogaert V, De Naeyer H, De Bacquer D, Ruige J, Kaufman JM, Taes YE. Birth Weight in relation to steroid status and body composition in young health male siblings. J Clin Endocrinol Metab. 2010, 95, 1587-1594.
23. Crabbe P, Bogaert V, De Bacquer D, Goemaere S, Zmierczak H, Kaufman JM. Part of the interindividual variation in serum testosterone levels in healthy men reflects differences in androgen sensitivity and feedback set point: contribution of the androgen receptor polyglutamine tract polymorphism. J Clin Endocrinol Metab. 2007, 92, 3604-3610.
24. Huhtaniemi IT, Pye SR, Limer KL, Thomson W, O'Neill TW, Platt H, Payne D, John SL, Jiang M, Boonen S, Borghs H, Vanderschueren D, Adams JE, Ward KA, Bartfai G, Casanueva F, Finn JD, Forti G, Giwercman A, Han TS, Kula K, Lean ME, Pendleton N, Punab M, Silman AJ, Wu FC; European Male Ageing Study Group. Increased estrogen rather than decreased androgen action is associated with longer androgen receptor CAG repeats. J Clin Endocrinol Metab. 2009, 94, 277-284.
25. American Diabetes Association. Standards of medical care in diabetes--2009. Diabetes Care. 2009 Jan;32 Suppl 1:S13-61.
26. Third Report of the National Cholesterol Education Program (NCEP) Expert Panel on Detection, Evaluation, and Treatment of High Blood Cholesterol in Adults (Adult Treatment Panel III) final report. National Cholesterol Education Program (NCEP) Expert Panel on Detection, Evaluation, and Treatment of High Blood Cholesterol in Adults (Adult Treatment Panel III). Circulation. 2002 Dec 17;106(25):3143-421
27. Vogeser M, Seger C. A decade of HPLC-MS/MS in the routine clinical laboratory--goals for further developments. Clin Biochem. 2008 Jun;41(9):649-62.
28. Vermeulen A, Verdonck L, Kaufman JM. A critical evaluation of simple methods for the estimation of free testosterone in serum.Clin Endocrinol Metab. 1999 Oct;84(10):3666-72.
29. Matthews DR, Hosker JP, Rudenski AS, Naylor BA, Treacher DF, Turner RC. Homeostasis model assessment: insulin resistance and beta-cell function from fasting plasma glucose and insulin concentrations in man. Diabetologia. 1985 Jul;28(7):412-9.
30. Schreiber D, Harlfinger S, Nolden BM, Gerth CW, Jaehde U, Schömig E, Klosterkötter J, Giuffrida A, Astarita G, Piomelli D, Markus Leweke F. Determination of anandamide and

other fatty acyl ethanolamides in human serum by electrospray tandem mass spectrometry. Anal Biochem. 2007 Feb 15;361(2):162-8.

31. Giagulli VA & MD Carbone. Varicocele correction for infertility: which patients to treat? Inter. J Androl, 33, 2010 (in press)

32. Dupont WD & Plummer WD: "Power and Sample Size Calculations: A Review and Computer Program", Controlled Clinical Trials 1990; 11:116-28

33. Dupont WD, Plummer WD: "Power and Sample Size Calculations for Studies Involving Linear Regression", Controlled Clinical Trials 1998; 19:589-601.

34. ICH Harmonized Tripartite Guidelines for Good Clinical Practice 1996 Directive 91/507/EEC; D.M. 15.7.1997
