# Supplementary material for: Clomiphene citrate effect in obese men with low serum testosterone treated with metformin due to dysmetabolic disorders: A randomized, double-blind, placebo-controlled study
Source: PLoS One. 2017 Sep 8;12(9):e0183369. doi: 10.1371/journal.pone.0183369 (PMC5590732; doi:10.1371/journal.pone.0183369)
Supplement: S2 File — English version of the protocol. (PDF) [file pone.0183369.s002.pdf]

ENGLISH VERSION

STUDY CODE: UOE/01-2011

Spontaneous interventional study title with drug: Effect of metformin and clomiphene in obese hypogonadal males with or without type 2 diabetes on plasma levels of testosterone and metabolic parameters

Authors: Renato Pasquali, Vito A. Giagulli
Phase. 3
Document: *Protocollo UOE/01001*
Version: final
Realese data: 26-01-2011

PROTOCOL SIGNED PAGE

Protocol CODE: UOE/01-2011

Renato Pasquali	firma	data
Vito A. Giagulli	firma	data
Antonio Maria Morselli Labate (Statistico)	firma	data

THE INVESTIGATOR'S STATEMENT:

Protocol CODE: UOE/01-2011

I have read the protocol and agree to conduct this clinical trial in accordance with all requirements of the Protocol in accordance with the Guidelines of Good Clinical Practice Guide and the principles of the Helsinki Declaration

Renato Pasquali	firma	data

INDEX:

1. Introduction
2. Aims of the study
3. Plan of the study
    3.1 Study design
    3.2 Study population
    3.3 Tretaments
    3.4. Visits and evaluations
    3.5. Efficacy assessments
    3.6. Safety assessments
    3.7 Genetic substudy
4. Data management and statistical analysis
    4.1. Data management
    4.2 Statistical methods 18
        - Characteristics of casistica , treatments and concomitant diseases
        - Evaluation of effectiveness: Primary variables
        - Evaluation of effectiveness: secondary variables
        - Statistical methods (efficiency rating)
        - Safety Rating
        - Sample size
5. Administrative procedures
6. References

1. Introduction

Several authors have recently reported that obese males (1, 2) and diabetes mellitus type 2 (T2DM) or with metabolic syndrome (MS) (3, 4, 5, 6), may result in reduced plasma levels of testosterone than those not suffering from these metabolic disorders. It is also known that the prevalence of obesity and diabetes is higher in patients in adulthood, and that the physiological aging in men is characterized by a slow and gradual decline in testicular function, revealed by a reduction in plasma levels of total testosterone (T) and free (FT) (7). Therefore it has been hypothesized that in the male the reduction in circulating levels of (F) T may play an important physiological role in aging, in insulin resistance and the risk of cardiovascular events genesis that follows (7, 8, 9) . Furthermore, it has been shown that the T in the male can positively modulate the insulin-sensitivity and incretin secretion independently of changes in body composition, as has recently been demonstrated in a group of normal undergo "subjects hyperinsulinemic euglycemic clamp" and variation of serum testosterone and serum estrogens by administering an aromatase inhibitor (letrozole) (10). It is generally accepted that in the male peripheral estradiol is the major factor that regulates the hypothalamus-pituitary secretion of gonadotropins, especially LH, and therefore plasma levels of (F) T (11,12). However the reduction in the aging male (F) T is not accompanied by a consensual reduction of 17 β estradiol (E2), which can even be increased in obese male (2). Recent studies have shown the efficacy of treatment with anti-estrogens and, in particular, of clomiphene citrate (CC) to increase the plasma levels of T in elderly males (14,15) in obese and hypogonadal subjects (13) or improving spermatogenesis in infertile males (16-19). The CC blocks the inhibitory feedback of estradiol on the hypothalamus, which increases the release of pituitary gonadotropins, LH and FSH, respectively stimulate the Leydig cells and Sertoli increasing the plasma testosterone levels and promoting spermatogenesis (20).

Therefore the aim of the study will evaluate the effects of treatment with clomiphene citrate (25 mg / day - Serofene) (CC) and metformin (2 g / day - Glucophage 1000) (MET), a drug used for the treatment of obese patients T2DM or impaired glucose tolerance, to the documented effectiveness in improving insulin sensitivity (21), on plasma levels of T in obese subjects, aged between 35 and 55 years, with ipogondismo and impaired glucose tolerance (IGT) or overt diabetes mellitus . Also it will study the effect of the expected increase of T on metabolic parameters comparing treatment with CC + MET compared to treatment with MET alone in all subjects enrolled in the study and divided according to the state of glucose tolerance.

This study will highlight the role of T in improving metabolic picture in obese hypogonadal subjects with impaired glucose tolerance or diabetes, opening new therapeutic perspectives.

2. Aims of the study

Primary objective of the study:

- To evaluate the effect of therapy with MET and CC on plasma testosterone levels.

Secondary objectives of the study:

- To evaluate the effect of therapy with MET and CC on:

1) plasma glucose levels, HbA1c, insulin and C-peptide, and insulin resistance index (HOMA-IR, homeostasis model assessment - insulin resistance)

2) other metabolic parameters (free fatty acids (FFA), total cholesterol, HDL, LDL, triglycerides);

3) circulating levels of markers of chronic inflammation (fibrinogen, CRP) and of inflammatory cytokines (TNF-a, IL-6);

4) plasma levels of the hormones secreted by adipose tissue (adiponectin, leptin);
5) plasma levels of endocannabinoids.

3. The Study Plan

3.1 Study design

The study is a randomized, double-blind, placebo-controlled, crossover, multicenter in a population of obese patients. The study, of the total duration of 30 weeks, is divided into a phase of selection of the population and a step of treatment in the cross-over with a wash-out period.
The subjects, stratified by the presence of diabetes mellitus (T2DM) or impaired glucose tolerance (IGT), will be randomized into two groups for the allocation to pharmacological treatment with metformin (MET), at a dose of 2000 mg per day, associated with clomiphene (CC) at a dose of 25 mg per day (treatment a), or metformin (MET) at a dose of 2000 mg a day plus placebo (PLAC) (treatment B).
The design in cross-over includes a first step of assigning to a treatment (A or B) for 12 weeks, followed by a period of drug wash-out 6 weeks after which the subjects will be assigned to the other treatment (B or A) (see diagram 1).
All patients will observe a low-calorie diet (1600 kcal / day) associated with moderate physical activity (25-30 minutes of walking a day) as recommended by the American Diabetes Association (25).

Scheme 1

**Phase**	Pre-randomization		Double-blind treatment				
**Period**	Selection	Basal	Tretament		Wash-out	Tretament	
**Week**	-1	0	6	12	18	24	30
**Visit**	V0	V1	V2	V3	V4	V5	V6
**Treatment**	None	None	MET + CC (A)<br>Oppure<br>MET + plac (B)			MET + CC (A)<br>Oppure<br>MET + plac (B)	

3.2 Study population

The study population will consist of 24 obese outpatients, including 12 suffering from type 2 diabetes, and 12 with impaired glucose tolerance (IGT). No subject should be in drug therapy for diabetes or other metabolic disorders defined in the metabolic syndrome (naïf patients).

Inclusion Criteria
The selected subjects must meet the following criteria:

- males between the ages of 35 and 55 years
- Obesity, defined according to the WHO criteria for a body mass index (BMI)>30 kg / m2
- Diagnosis of diabetes or impaired glucose tolerance according to the criteria defined by the American Diabetes Association (ADA) in 2009 (25): subjects with blood sugar at the second hour after glocosio oral tolerance test (OGTT) in particular will be considered to have impaired glucose tolerance between 140 and 199 mg / dl, and diabetic subjects with fasting blood glucose> 126 mg / dl or blood glucose at the second hour after glocosio oral

tolerance test (OGTT)> 200 mg / dl

- HbA1c < 8.5%
- Metabolic syndrome, defined according to the ATP III guidelines, or for the simultaneous presence of 3 or more of the following disorders: central obesity (waist circumference ≥ 102 cm); high blood pressure (SBP ≥ 130 mmHg or PAD ≥ 85 mmHg or drug therapy for high blood pressure); hypertriglyceridemia (≥ 150 mg / dL or drug therapy hypertriglyceridemia); low HDL cholesterol (<40 mg / dL or drug therapy for low HDL cholesterol) (26)
- hypogonadism, defined for circulating levels of total testosterone ≤ 3 ng / ml (7)
- signing of informed consent.

Exclusion criteria

- Patients with primary or secondary hypogonadism in nature associated with genetic diseases or infiltrative or destructive processes borne by the endocrine organs (testicles, hypothalamus-pituitary)
- Patients on drug therapy with oral hypoglycemic agents in place or in the 3 months prior to the start of the study
- Patients with lipid-lowering drug therapy in place or in the 3 months prior to the start of the study
- Known or suspected hypersensitivity to the drug or to drug class in the studio;
- Patients with serious medical conditions that, in the opinion of the investigator, contraindicate the patient's participation in the study;
- Use of experimental drugs systemically over the last three months prior to study entry.
- Patients unable to follow the procedures of the Protocol.

3.3 Treatments

Study treatments

Metformin will be supplied as tablets of 1000 mg in the form of packs already commercially available. Metformin will be administered at a dose of 1000 mg, 2 times a day, taken during or after meals.

Clomiphene, supplied in tablets of 25 mg Serofene by manufacturer Merck Serono will be set as 25 mg capsules from pharmaceutics Laboratory of Hospital Pharmacy of the Hospital of Bologna.

Clomiphene will be administered at a dose of 25 mg, once a day. The placebo will be set up by Famacia Hospital in the same pharmaceutical form of clomiphene, and administered once a day.

Treatment assignment

Confirmed the eligibility, a unique identifier will be assigned by the investigator to each trial center to the patient in the study. The code is the number of the center followed by the consecutive number of entry into the study at the experimental center. Once assigned, the codes of patients who for any reason do not continue the study can no longer be reused

Patients meeting the inclusion and exclusion criteria will be randomized to one of the study treatments (Treatment A versus treatment B).

The assignment of the patient to one of the treatment groups takes place according to the randomization list generated through computer Investigational Drug Service (IDS) at the Pharmacy of the Coordinating Center Hospital respecting the double-blind criteria.

The randomization list will be forwarded, in the part of them, in the hospital pharmacists identified as a reference for the study in each experimental center and may not be disclosed dutrante the course of the trial to other investigators involved in the clinical trial in question. Following randomization request, submitted by the investigators to their

contact pharmacist, the hospital pharmacy of each center assigns a random number, prepares the experimental drug / placebo and sends it to the requesting investigator ensuring double blindness.

The patient will be assigned the randomization code more 'lowest available.

If during the study you will be necessary to know the code of treatment of a patient, see in Ch. 5 (administrative procedures) the procedure for opening the emergency codes. The opening of the emergency code is allowed in exceptional cases only to take appropriate therapeutic measures otherwise unidentifiable.

Concomitant therapies

Will not be permitted during the study the use of the following drugs:

- oral hypoglycemic agents other than metformin and other antidiabetic agents
- of glucocorticoid systemic synthesis
- hormone therapy (androgens, antiandrogens)
- lipid-lowering drugs.

Use will be allowed during the study for:

- lipid-lowering medication if LDL-cholesterol is not in the target after 6 months from the start of the study (LDL> 100 mg / dl)
- anti-hypertensive drugs.

Treatment discontinuation

Each patient has full authority to interrupt his participation in the study at any time; Also, if you believe that it is beneficial to one's health, the patient's participation in the study will be interrupted. In particular in case of need to add a second drug therapy oral hypoglycemic agent to inadequate metabolic control (HbA1c ≥ 8.5%) will stop the patient's participation in the study.

Adherence to treatment

Patients will be asked to deliver at every visit and at the end of the study all study drugs not used. The amount of returned medications must be documented. The investigator will check the patient's adherence to the prescribed dose and will act appropriately in case of non-compliance.

In case of interruption of the experimental treatment lasting more than 7 consecutive days, the patient will be excluded from the study and will not continue with the experimental treatment (drop-out).

3.4. Visits and assessments

Visit selection

Before starting any specific procedure of the study, the researcher must obtain the patient's written informed consent signed. The screening assessments include blood chemistry and hormonal laboratory tests, an oral glucose tolerance test and a clinical examination. Only for eligible patients the feedback will be reported in CRF at the V0 visit.

Scheme for medical examinations and assessments

The number of total visits 7 (V0-V6, according to the assessments diagram). The procedures regarding individual surveys shall be recorded in the assessments scheme. These visits could be deferred / postponed by seven days.

Evaluations scheme

Numeber of Visit	V0	V1	V2	V3		V4	V5	V6
**Treatment**	Selezione	Randomizzazione (assegna-zione A o B)	A o B	A o B	Wash-out	Cross-over (assegna-zione B o A)	B o A	B o A
**Period**	giorni – 7/0	sett. 0 ±7 gg	sett. 6 ±7 gg	sett. 12 ±7 gg		sett. 18 ±7 gg	sett. 24 ±7 gg	sett. 30 ±7 gg
**Informed Consent**	X							
**Clinical history**<br>**Therapy used**	X<br>X	X<br>X				X<br>X		
**Cncomitant therapy**	X	X	X	X		X	X	X
**Physicla examination**<br>**Questionarie qADAM, CDQ, CSD, Androtest, IIEF**		X<br>X	X	X<br>X		X<br>X	X	X<br>X
**Lab test**	X			X		X		X
**Diet interview**		X				X		
**Inclus. Excl. Criteria**	X	X						
**Drug delivery**		X				X		
**Adverce events**		X	X	X		X	X	X
**Assesment of satisfaction degrre for the treatment**				X				X

A: tretament A (CC+MET); B: tretament B (PLAC+MET)

Laboratory tests

Screening will be carried out the blood chemistry and hormonal tests under fasting conditions. In particular screening all individuals carry out a baseline blood sample for the determination of:

- metabolic parameters (blood glucose, insulin, c-peptide, HbA1c, free fatty acids (FFA), total cholesterol, HDL, LDL, triglycerides, GOT, GPT, GGT, alkaline phosphatase, protide my total and fractionated, creatinine);
- markers of chronic inflammation (fibrinogen, CRP) and inflammatory cytokines (TNF-a, IL-6);
- hormones and proteins (total testosterone and SHBG, TSH, FT4);
- oral glucose tolerance test (OGTT) with 75 g of glucose with glucose dose, insulin and C-peptide (time 0 ', 30'. 60 ', 90', 120 ').

You will also be taken aliqote blood and serum for the dosage of hormones secreted by adipose tissue (adiponectin, leptin) and endocannabinoids.

The examinations listed above will be made at the time of the visit V0, V3, V4 and V6.

The OGTT shall only be V0 to define the state of glucose tolerance and inclusion in the study.

The aliquots of serum and plasma and whole blood of each subject will be stored in a freezer at -80 ° C until the

analytical determination; analyzes on samples of all subjects will be made at the end of the study to avoid intra-assay variations. They will also be determined levels of testosterone by HPLC-MS / MS method (high-performance liquid chromatography with tandem mass spectrometry) (27), while the FT will be calculated using the formula of Vermeulen (28). The plasma levels of SHBG will be measured by immunoassay method, insulin and C-peptide vennaro measured ECLIA method, PCR method Turbidimetric / immunonefel., Total cholesterol by CHOD-PAP method, enzymatic method with homogeneous colorimetric HDL, triglycerides with GPO- method PAP, FFA with ACS ACOD method, IL-6 with chemiluminescence, and TNF-α immunoassay. As insulin resistance index HOMA-IR will be made the calculation according to Matthews (29). For the dosage of adiponectin and leptin we will be using the RIA method (kit for leptin: Millipore HL-81HK, for adiponectin Millipore HadP-61HK). For the determination of endocannabinoids we will be using the HPLC-MS / MS method (30).
Determinations will be made at the Laboratory Centralizzatro the S. Orsola-Malpighi and at the Research Centre of Applied Biology, University of Bologna.

3.5. Efficacy assessments

The primary objective of the study is to evaluate the effect of therapy with metformin and clomiphene on plasma testosterone levels.

3.6. Safety assessments

The safety assessment of the drug will consist in monitoring and recording of adverse events, of serious adverse events, laboratory examination and measurement of vital signs.

Adverse events

Information related to all adverse events, both those reported spontaneously by the subject and those encountered by the Investigator as a result of specific questions that those evidence physical examination of the patient, from laboratory investigations or otherwise, will be collected, recorded on the CRF and followed as appropriate.
It defines adverse event each sign, symptom or adverse clinical condition that occurs after the start of treatment with the drug (or therapy) administered, although there is no causal relationship between the event and the drug (or therapy) administered. The drug (or therapy) administered includes medication (or treatment) being measured and each drug (or therapy) comparison or placebo, administered during any phase of the study.
The clinical conditions / diseases already present before the start of treatment with the drug administered adverse events are considered only if they get worse after starting treatment with the drug administered. Any anomalies detected by laboratory tests or tests are adverse events only if they cause signs or symptoms, if they are considered to be relevant from a clinical point of view or if you require therapy, and are recorded in the CRF following the signs, symptoms or diagnosis associated.
As far as possible, any adverse event is described in terms of the level of severity (mild, moderate, severe) (levels 1-3).

Serious Adverse Events

The information for all serious adverse events will be collected and recorded on the reporting form of Serious Adverse Events. To ensure patient safety, all serious adverse events must be reported within 24 hours of the investigator becomes aware.

Adverse events are defined serious those adverse events that:

- They are fatal;
- place the patient in danger of life;
- require patient hospitalization or prolong hospitalization;
- involve disability / significant and persistent inability although not necessarily permanent;
- They are significant from a medical point of view in that it can harm the patient and require medical or surgical intervention to prevent situations listed above.

They are not considered serious adverse events any hospitalizations:

- routine treatment or monitoring of the clinical condition of the object of observation, not associated with worsening of the clinical condition itself;
- elective treatments or pre-scheduled for pre-existing medical conditions that are not related to the pathology observed and who have not deteriorated;
- General treatment (in hospital or other institutions) not associated with any worsening of general clinical conditions;
- Emergency treatments on an outpatient basis for events that do not meet the definition of serious adverse events and no fee to enter the hospital.

Must also be given any serious adverse event that occurs after the patient has given informed consent and until 4 weeks after the last dose of study drug. Serious adverse events that occur after four weeks have elapsed since the patient has taken the last dose of study medication, should only be reported if you suspect a causal relationship to medication (or therapy) administered during the study .

The Investigator must complete the reporting form of serious adverse events, assess the causal relationship to the study drug.

Any recurrent episode, complications or progression of an event already reported must be reported as a follow-up to that event.

The information follow-up must be notified with a new card, specifying that it is the follow-up to an event already reported and indicating the date of the original notification took place.

Contact Persons

The telephone and fax numbers of the contact persons for reporting Serious Adverse Events were reported nell'Investigator Folder.

3.7 Genetic substudy

Plasma concentrations of total testosterone (T) and its free quota (FT) have a considerable interindividual variability. There are environmental factors (obesity, smoking, etc.) (21) and genetic factors (22) able to influence the circulating levels of testosterone. Studies on large populations of healthy male subjects have shown that the inter-individual variability in FT concentrations reflect differences in androgen sensitivity. In particular a direct correlation between the concentrations of the FT and the length of the CAG repeat in exon 1 of the androgen receptor gene (AR) gene coding a

polymorphic glutamine tract (23,24) has been documented.

Therefore, the CAG polymorphism of androgen receptor in obese subjects with impaired glucose tolerance or overt diabetes may explain individual differences in therapeutic response, especially on metabolic picture, despite the expected increase in testosterone concentrations.

All subjects participating in the study will be offered participation in the genetic substudy that involves the determination of the CAG polymorphism of the androgen receptor.

The CAG polymorphism in the first exon of the androgen receptor is measured by the method described in the literature (31) at the Center for Genetics and Molecular Biology of the clinical research center and Hormonal "Telesforo" (Via Rosati, 137 / C, Foggia, responsible Laboratory Dr. Dominic Carbone). From a sample of 300 mcL of samgue will be extracted DNA (AB Analytical Ltd., Advance Biomedicine, REF. 05-42, Padova, Italy), which will be amplified with specific primers with polymerase method (PCR). The counts of CAG will be made through an automatic analyzer [ABI PRISM 3100 Genetic Analyzer (Perkim-Elmer Cop.)]. The determination of the length of the CAG (260-320 bp) will be carried out twice in two separate determinations.

4. Data management and statistical analysis

4.1. Data management

The staff designated by the investigator must contain the information required by the Protocol on the Data Collection Sheet (CRF).

The data of the CRF will be entered centrally by personnel designated by the Investigator through single data entry with electronic verification of data. The text elements (eg. Comments) will be verified manually. The data will be subsequently checked by the validation and control of programs listed. Obvious errors will be corrected directly from the data management staff, other errors or omissions will be verified on the medical records.

Information regarding concomitant medications will be coded using the WHO Drug Reference List, which uses the classification system Anatomic Therapeutic Chemical (ATC). Comorbidities and adverse events will be coded using the terminology of ICD9 classification.

The database will be closed once complete and accurate it said. Any change to the next data to the closure may only be made with the written agreement of the clinician responsible for the spontaneous study.

4.2 Statistical methods

The aim of this study is to evaluate the efficacy of treatment with MET and CC in increasing testosterone levels in obese subjects with IGT and T2DM compared to with MET treatment and PLAC.

The data collected will be grouped and summarized with respect to demographic variables, the baseline characteristics and the efficacy and safety assessments.

The exploratory analyzes will be performed using descriptive statistics:

- Means, standard deviations, absolute and relative frequencies, range, confidence intervals.

The data will be presented for both the intent-to-treat population (ie all patients who took at least one dose of study) that for the per-protocol population (ie all patients who completed the study without major violations of protocol).

The safety assessment will be mainly based on the frequency of adverse events, including all serious adverse events. Adverse events will be summarized for each treatment group by presenting the number and percentage of patients who experienced any adverse event, an adverse event in a specific organism apparatus and a specific adverse event. Any other information collected (i.e. the severity or relationship to study drug) will be encoded as appropriate.

We will also be produced analytical lists which contain more information regarding:

- Patients who discontinued the study and the reasons therefor;
- Patients who discontinued the study due to adverse events;
- Patients who experienced serious adverse events;
- Patients with values of laboratory tests outside the predefined ranges.

Characteristics of casistica , treatments and concomitant diseases

Data on demographic and baseline characteristics and observations of efficacy and safety measures will be appropriately summarized. The characteristics of administration of the study drug and other concomitant treatments will be summarized, like the main concomitant diseases recorded on admission to the study. They will also be listed patients who discontinued the study and described in detail its reasons.

The exploratory analyzes will be performed using descriptive statistics by dividing patients by type of treatment, the treatment sequence and glucose tolerance.

Will be reported: means, standard deviations (SD), absolute and relative frequencies, range, confidence intervals.

Evaluation of effectiveness: Primary variable

The population on which will be performed the primary analysis of efficacy and 'consists of all randomized patients who have received both treatments.

The primary efficacy variable is the increase in plasma testosterone levels after treatment.

Evaluation of effectiveness: secondary variables

The secondary efficacy variables are improved metabolic control, defined by reduced levels of blood glucose and HbA1c, insulin and C-peptide, reduction of insulin resistance index (HOMA-IR), lipid improvement (reduction of levels total cholesterol, LDL and triglycerides and increased HDL levels, reduced levels of FFA), reduction of chronic inflammation (fibrinogen, CRP) and inflammatory cytokines (TNF-α, IL-6) after treatment.

Statistical methods (efficiency rating)

The normality of the data will be verified using ol Kolmogorov-Smirnov test.

The data will be evaluated using analysis of variance (ANOVA) for repeated measures three-way (type of treatment, sequence of treatment, glucose tolerance).

Safety Rating

The population of which it will be carried out the security analysis and 'the' Safety Population 'consists of all patients included in the study who have received at least one dose of study medication.

Adverse events will be summarized by presenting the number and percentage of patients with any type of event, classified by body system and by preferred term. Other information collected (eg. Severity, causal relationship) will be penciled well as patients with serious adverse events.

Will also be listed patients had serious adverse events and discontinued the study due to adverse events.

The values of the laboratory tests will be summarized in the appropriate tables that will bring the frequency of the values outside of a predetermined range. The values that represent significant abnormalities will be listed.

Other safety data (eg. Vital signs and specific tests) will be evaluated appropriately.

Sample size

The study and 'was designed to demonstrate that the effect of the association of metformin and clomiphene' higher than the effect of metformin on testosterone levels after 12 weeks of treatment.

E 'was calculated that to have a probability of at least 80% to find, with a significance level of 5% (two-tailed), a difference between treatments of 2.8 ng / mL of testosterone level with DS of 1.5 ng / mL, a total of 20 patients (28,29) must be assessed. Therefore it was decided to randomize 24 patients in order to compensate for a drop-out rate of the order of 10-20%.

The sample size was calculated with the program "PS Power and Sample Size Calculations" of the Department of Statistics of Vanderbilt University, Nashville, TN, USA (Version 3.0.12; http://biostat.mc.vanderbilt.edu/twiki / bin / view / Main / PowerSampleSize) using the steps of Dupont and Plummer (28, 29).

5. Administrative procedures

Good Clinical Practice standards

This study will be conducted in accordance with the principles of Good Clinical Practice (30), with the Declaration of Helsinki and national regulations regarding the conduct of clinical trials. The experimenter, by signing the Protocol, agrees to adhere to the procedures and the instructions contained therein and to conduct the study in accordance with GCP, the Declaration of Helsinki and the national regulations governing clinical trials.

Amendments to the Protocol or any other amendments to the conduct of the study

Any changes to the protocol will be made as an amendment. Any modification to the protocol during the study period. Any unexpected change in the conduct of the trial will be recorded in "Clinical Study Report."

Ethics committees and informed consent

The study protocol, any amendment of the protocol, informed consent and any other information for patients must be approved by the Ethics Committee of the structure where it operates the Investigator.

As regards the amendments, the investigator can immediately apply them by written notice to the Ethics Committee, without waiting for the approval of the Ethics Committee, if the safety of patients participating in the study is at stake. Also, if the investigator believes that patient safety reasons it is necessary to immediately make a change to the protocol, it must put aware the Ethics Committee of the center within 10 working days.

To participate in the study each patient will have to give written informed consent (see also section 3.2 - Study population).

Documentation Repository

The investigator is responsible for filing and storage of essential documents of the study, before, during conducting and after the completion or termination of the study, according to the / and for the time provided by law and by GCP.

The data collected on the CRF will be strictly anonymous and the subject will be solely identified with a number and the initials.

The investigator must keep the patient's original data (eg. Demographic and medical information, laboratory data, electrocardiograms etc.) and a copy of the written informed consent signed. For some data may be established, before the beginning of the study, which are written directly on the CRF, which thus in this case will act as the original data.

Emergency procedures for the suspension of blindness

Simultaneously sending the randomization list, which will be forwarded to hospital pharmacists identified as a reference

for the study in each experimental center and which can not be disclosed dutrante the course of the trial to other investigators involved in the clinical trial in question, it will be forwarded to the investigator of each experimental center individual sealed envelopes corresponding to the part of the randomization list for specific experimental center. Each envelope identified with the randomisation number containing information on (drug / placebo) type of treatment given to the patient. The envelope will be sigilata apèerta by the Investigator of each individual only in an emergency center. The Speirmantatore must at the same time documenting the reason of the opening of the code, the date / time of the opening of the envelope and the contenutop document in a sealed envelope.
The investigator shall also immediately notify the head of the study..

Inspection / Audits

The Investigator or delegated staff will conduct inspections during the study to ensure that it is conducted in accordance with the Protocol and the applicable regulatory requirements. Also regulatory agencies can conduct inspections on the study (during it or after the study was completed). If the Regulatory Authority requires an inspection, the investigator must immediately inform the head of the study. By signing the protocol, the investigator agrees both to audits by der study leader or deputy staff inspections of the Regulatory Authority.

Medication management study

The study drug will be provided entirely by the Investigator.

Metformin will be supplied as tablets of 1000 mg in packs already commercially available.

Clomiphene, supplied by the manufacturer Merck Serono as Serofene tablets of 25 mg, will be set up by the Company's Pharmacy Hospital-University of Bologna as 25 mg capsules for administration to the patient.

The investigator must ensure that the study drug is used in accordance with the protocol.

The investigator is responsible for maintaining the medication in a secure place, with limited access. The drug must be maintained in accordance with the storage conditions on the package.

Procedure for the preparation of the experimental sample in pharmacies
The Company's Pharmacy University Hospital of Bologna organizes experimental medicines pursuant to and in compliance with the requirements of Article 15 of Legislative Decree 06/11/2007 n. 200 in accordance with the requirements of Good Preparation Medicines, according to the current edition of the Italian Pharmacopoeia, to ensure the quality, effectiveness and safety of medicinal product prepared.
The Galenic Laboratory of Hospital Pharmacy of the Hospital of Bologna, will set the placebo capsules (rice starch) and 25 mg clomiphene.

Method of preparation of capsules of 25 mg or placebo Clomiphene

It is ground, in mortar Login, the tablets of Serofene from 25 mg up to obtain a fine powder.

The clomiphene powder thus obtained and possible excipients and placebo, the powder of rice starch are mixed according to the method of progressive dilutions and make it homogeneous by mixing. If they are present in a reduced quantity can be convenient to add an inert diluent excipient to increase the volume and to allow for statistically reduce the possibility of error during the subdivision.

The experimental drug or placebo will be prepared for lots with un'opercolatrice and automatic guiding tray with 300 capsules.

The validity of the product is 6 months at room temperature with a possible option to extend it for one year on the basis of specific assessments by the trainer pharmacist.

The preparation will be documented on special register dedicated to the clinical trials on which are recorded the n. Batch, date of preparation, the quantitative composition, the validity, the discharge papers from the drug store, the signature of the preparer pharmacist, suitability for distribution of the finished product.

The capsules of experimental / placebo medication will then be packed in special polyethylene containers and labeled.

In addition, prior to deployment, the Pharmacist in charge will perform the following checks:

- Verification of the correctness of the procedures performed
- Controlling the appearance and the capsule sealing
- Monitoring the number of arranged capsules and the quantity to be dispensed
- Control of the primary and secondary packaging
- Verification of the correct label compilation
- Essay mass uniformity on the lot.

Publication of results

Any formal presentation or publication of data derived from this study is intended as a joint publication by the investigator and head of the study. For multi-center studies, it is mandatory that the first publication is based on the data of all the centers, analyzed according to the protocol. Investigators participating in multi-center studies agree not to submit data from a single center or a small group of centers unless there is a formal agreement by the other experimenters and Dr. Giagulli VA (proposer of the spontaneous study) that must receive a copy of every communication that will be published in advance of the publication itself.

Confidentiality and Privacy

The study documents submitted by the investigator (eg. The protocol, CRF and other) must be kept in a safe place to ensure the maintenance of confidentiality and privacy. The information and documents submitted by Sperimentatopre will not be disclosed to others without written permission of the head of the study, except to the extent necessary to obtain the patient's consent to study participation.

Study Termination

The study manager reserves the right to stop the study in the case of serious adverse events associated with intake of the experimental treatment in two or more subjects recruited.

Contact Persons

The telephone and fax numbers of the contact persons for the conduct of the study are reported nell'Investigator Folder.

6. References

1. Zumoff B, Strain GW, Miller LK, Rosner W, Senie R, Seres DS, Rosenfeld RS. Plasma free and non-sex-hormone-binding-globulin-bound testosterone are decreased in obese men in proportion to their degree of obesity. J Clin Endocrinol Metab. 1990 Oct;71(4):929-31.

2. Giagulli V.A., Kaufman JM, Vermeulen A. Pathogenesis of the decreased androgen levels in obese men. J Clin Endocrinol Metab. 1994 Oct;79(4):997-1000.

3. Pitteloud N, Mootha VK, Dwyer AA, Hardin M, Lee H, Eriksson KF, Tripathy D, Yialamas M, Groop L, Elahi D, Hayes FJ. Relationship between testosterone levels, insulin sensitivity, and mitochondrial function in men. Diabetes Care. 2005 Jul;28(7):1636-42.

4. Ding EL, Song Y, Malik VS, Liu S. Sex differences of endogenous sex hormones and risk of type 2 diabetes: a systematic review and meta-analysis. JAMA. 2006 Mar 15;295(11):1288-99.

5. Chen RK et al. Diab.obes.Met 8(4) 4292006

6. Grossmann M, Thomas MC, Panagiotopoulos S, Sharpe K, Macisaac RJ, Clarke S, Zajac JD, Jerums G. Low testosterone levels are common and associated with insulin resistance in men with diabetes. J Clin Endocrinol Metab. 2008 May;93(5):1834-40.

7. Kaufman JM & Vermeulen A. The decline of androgen levels in elderly men and its clinical and therapeutic implications. Endocr Rev. 2005 Oct;26(6):833-76.

8. Hak AE, Witteman JC, de Jong FH, Geerlings MI, Hofman A, Pols HA. Low levels of endogenous androgens increase the risk of atherosclerosis in elderly men: the Rotterdam study. J Clin Endocrinol Metab. 2002 Aug;87(8):3632-9.

9. English KM, Mandour O, Steeds RP, Diver MJ, Jones TH, Channer KS. Men with coronary artery disease have lower levels of androgens than men with normal coronary angiograms. Eur Heart J. 2000 Jun;21(11):890-4.

10. Lapauw B, Ouwens M, 't Hart LM, Wuyts B, Holst JJ, T'Sjoen G, Kaufman JM, Ruige JB. Sex steroids affect triglyceride handling, glucose-dependent insulinotropic polypeptide, and insulin sensitivity: a 1-week randomized clinical trial in healthy young men. Diabetes Care. 2010 Aug;33(8):1831-3.

11. T'Sjoen GG, Giagulli VA, Delva H, Crabbe P, De Bacquer D, Kaufman JM. Comparative assessment in young and elderly men of the gonadotropin response to aromatase inhibition. J Clin Endocrinol Metab. 2005 Oct;90(10):5717-22.

12. Raven G, de Jong FH, Kaufman JM, de Ronde W. In men, peripheral estradiol levels directly reflect the action of estrogens at the hypothalamo-pituitary level to inhibit gonadotropin secretion. J Clin Endocrinol Metab. 2006 Sep;91(9):3324-8.

13. Loves S, Ruinemans-Koerts J, de Boer H. Letrozole once a week normalizes serum testosterone in obesity-related male hypogonadism. Eur J Endocrinol. 2008 May;158(5):741-7.

14. Leder BZ, Rohrer JL, Rubin SD, Gallo J, Longcope C. Effects of aromatase inhibition in elderly men with low or borderline-low serum testosterone levels. J Clin Endocrinol Metab. 2004 Mar;89(3):1174-80.

15. Veldhuis JD, Iranmanesh A. Short-term aromatase-enzyme blockade unmasks impaired feedback adaptations in luteinizing hormone and testosterone secretion in older men. J Clin Endocrinol Metab. 2005 Jan;90(1):211-8.

16. Duschek EJ, Gooren LJ, Netelenbos C. Comparison of effects of the rise in serum testosterone by raloxifene and oral testosterone on serum insulin-like growth factor-1 and insulin-like growth factor binding protein-3. Maturitas. 2005 Jul 16;51(3):286-93.

17. Vandekerckhove P, Lilford R, Vail A, Hughes E. WITHDRAWN: Androgens versus placebo or no treatment for

idiopathic oligo/asthenospermia. Cochrane Database Syst Rev. 2007 Jul 18;(4):CD000150. Review.

18. Ghanem H, Shamloul R. An evidence-based perspective to the medical treatment of male infertility: a short review. Urol Int. 2009;82(2):125-9.

19. Kaminetsky J, Hemani ML. Clomiphene citrate and enclomiphene for the treatment of hypogonadal androgen deficiency. Expert Opin Investig Drugs. 2009 Dec;18(12):1947-55.

20. Goldstein SR, Siddhanti S, Ciaccia AV, Plouffe L Jr. A pharmacological review of selective oestrogen receptor modulators. Hum Reprod Update. 2000 May-Jun;6(3):212-24.

21. Kaufman JM, Vermeulen A. The decline of androgen levels in elderly men and its clinical and therapeutic implications. Endocr Rev. 2005, 26, 833-876.

22. Vanbillemont G, Lapauw B, Bogaert V, De Naeyer H, De Bacquer D, Ruige J, Kaufman JM, Taes YE. Birth Weight in relation to steroid status and body composition in young health male siblings. J Clin Endocrinol Metab. 2010, 95, 1587-1594.

23. Crabbe P, Bogaert V, De Bacquer D, Goemaere S, Zmierczak H, Kaufman JM. Part of the interindividual variation in serum testosterone levels in healthy men reflects differences in androgen sensitivity and feedback set point: contribution of the androgen receptor polyglutamine tract polymorphism. J Clin Endocrinol Metab. 2007, 92, 3604-3610.

24. Huhtaniemi IT, Pye SR, Limer KL, Thomson W, O'Neill TW, Platt H, Payne D, John SL, Jiang M, Boonen S, Borghs H, Vanderschueren D, Adams JE, Ward KA, Bartfai G, Casanueva F, Finn JD, Forti G, Giwercman A, Han TS, Kula K, Lean ME, Pendleton N, Punab M, Silman AJ, Wu FC; European Male Ageing Study Group. Increased estrogen rather than decreased androgen action is associated with longer androgen receptor CAG repeats. J Clin Endocrinol Metab. 2009, 94, 277-284.

25. American Diabetes Association. Standards of medical care in diabetes--2009. Diabetes Care. 2009 Jan;32 Suppl 1:S13-61.

26. Third Report of the National Cholesterol Education Program (NCEP) Expert Panel on Detection, Evaluation, and Treatment of High Blood Cholesterol in Adults (Adult Treatment Panel III) final report. National Cholesterol Education Program (NCEP) Expert Panel on Detection, Evaluation, and Treatment of High Blood Cholesterol in Adults (Adult Treatment Panel III). Circulation. 2002 Dec 17;106(25):3143-421

27. Vogeser M, Seger C. A decade of HPLC-MS/MS in the routine clinical laboratory--goals for further developments. Clin Biochem. 2008 Jun;41(9):649-62.

28. Vermeulen A, Verdonck L, Kaufman JM. A critical evaluation of simple methods for the estimation of free testosterone in serum.Clin Endocrinol Metab. 1999 Oct;84(10):3666-72.

29. Matthews DR, Hosker JP, Rudenski AS, Naylor BA, Treacher DF, Turner RC. Homeostasis model assessment: insulin resistance and beta-cell function from fasting plasma glucose and insulin concentrations in man. Diabetologia. 1985 Jul;28(7):412-9.

30. Schreiber D, Harlfinger S, Nolden BM, Gerth CW, Jaehde U, Schömig E, Klosterkötter J, Giuffrida A, Astarita G, Piomelli D, Markus Leweke F. Determination of anandamide and other fatty acyl ethanolamides in human serum by electrospray tandem mass spectrometry. Anal Biochem. 2007 Feb 15;361(2):162-8.

31. Giagulli VA & MD Carbone. Varicocele correction for infertility: which patients to treat? Inter. J Androl, 33, 2010 (in press)

32. Dupont WD & Plummer WD: "Power and Sample Size Calculations: A Review and Computer Program", Controlled Clinical Trials 1990; 11:116-28

33. Dupont WD, Plummer WD: "Power and Sample Size Calculations for Studies Involving Linear Regression",

Controlled Clinical Trials 1998; 19:589-601.

34. ICH Harmonized Tripartite Guidelines for Good Clinical Practice 1996 Directive 91/507/EEC; D.M. 15.7.1997
