# Supplementary material for: Clomiphene citrate effect in obese men with low serum testosterone treated with metformin due to dysmetabolic disorders: A randomized, double-blind, placebo-controlled study
Source: PLoS One. 2017 Sep 8;12(9):e0183369. doi: 10.1371/journal.pone.0183369 (PMC5590732; doi:10.1371/journal.pone.0183369)
Supplement: S3 File — Detalied explanation of the sample size determination. (DOC) [file pone.0183369.s003.doc]

**SUPPORTING DATA SAMPLE SIZE**

Unfortunately, a mistake occurred in reporting the SD value in the original study protocol. In fact, the hypothesized value of 1.5 ng/mL is unlikely and an unequivocally inappropriate sample size would result by taking into account that value (4 patients).

*Following you can find the original procedure applied for the sample size determination*

The trial was designed in order to show the superior efficacy of CC+MET treatment in increasing T levels in comparison with the Plac+MET treatment. As hypothesized mean difference of the modification of T between treatments was chosen a value equal to the lower hormonal limit of the normal range for T level in healthy young men generally reported by the most laboratories and in agreement with the statement of several international societies in the field aiming at defining the male hypogonadims (i.e., 2.8 ng/mL) [Wang C1, et al. Eur J Endocrinol. 2008; Bhasin S et al. J Clin Endocrinol Metab 2010]. In order to estimate the SD values we took into account the SD data regarding age specific modification of T in healthy males subjects after CC treatment reported by Tenover JS *et al*. [JCEM 1987]. They studied age spans lower (22-35 years) and higher (65-84 years) in comparison to the age range of our study (35-55 years) and they found values of standard deviation (SD) equal to 3.8 and 2.6 ng/mL in the lower and higher age span, respectively. Since no data regarding the within-subject variability of T was available, in the literature, the sample size was computed conservatively by considering an independent sample design (i.e., by using the SD within group) instead of a cross-over design (i.e., by using the SD of the changes). The hypothesized value of the SD for the calculation of sample size of our study was estimated as the mean value obtained by weighting 1 and 2 the values of the lower and higher age span groups of the Tenover JS *et al* data (i.e., 3.0 ng/mL) in order to better simulate the age span of our study population. The power analysis revealed that a number of 19 subjects would be required in order to achieve a power of 80% and a type I error of 5%; thus, we decide to randomize 24 patients in order to allow us to compensate for a possible frequency of drop-out of about 20%. The sample size was evaluated by means of the “PS Power and Sample Size Calculations” of Department of Statistics, Vanderbilt University, Nashville, TN, USA (Version 3.0.12, [http://biostat.mc.vanderbilt.edu/wiki/Main/PowerSampleSize#PS:_Power_and_Sample_Size_Calculation](http://biostat.mc.vanderbilt.edu/wiki/Main/PowerSampleSize#PS:_Power_and_Sample_Size_Calculation)) [Dupont WD & Plummer WD 1990, 1998].

**References**

Wang C1, Nieschlag E, Swerdloff R, Behre HM, Hellstrom WJ, Gooren LJ, Kaufman JM, Legros JJ, Lunenfeld B, Morales A, Morley JE, Schulman C, Thompson IM, Weidner W, Wu FC. Investigation, treatment and monitoring of late-onset hypogonadism in males: ISA, ISSAM, EAU, EAA and ASA recommendations. Eur J Endocrinol. 2008;159:507-14. doi: 10.1530/EJE-08-0601

Bhasin S, Cunningham GR, Hayes FJ, Matsumoto AM, Snyder PJ, Swerdloff RS, Montori VM; Task Force, Endocrine Society. Testosterone Therapy in Men with Androgen Deficiency Syndromes: An Endocrine Society Clinical Practice Guideline J Clin Endocrinol Metab 2010: 95: 2536-2559.

Tenover JS Matsumoto AM, Plymate SR, Bremner WJ. The effects of aging in normal men on bioavailable testosterone and luteinizing hormone secretion: response to clomiphene citrate J Clin Endocrinol Metab. 1987;65:1118-26.

Dupont WD & Plummer WD. Power and sample size calculations. A review and computer program. Control Clin Trials. 1990;11:116-28.

Dupont WD & Plummer WD Power and sample size calculations for studies involving linear regression. Control Clin Trials. 1998;19:589-601.
